# Understanding how environmental enhancement and conservation activities may benefit health and wellbeing: a systematic review

**DOI:** 10.1186/s12889-015-2214-3

**Published:** 2015-09-07

**Authors:** Rebecca Lovell, Kerryn Husk, Chris Cooper, Will Stahl-Timmins, Ruth Garside

**Affiliations:** European Centre for Environment and Human Health, University of Exeter Medical School, RCHT, Truro, TR1 3HD UK; NIHR CLAHRC South West Peninsula (PenCLAHRC), Plymouth University Peninsula Schools of Medicine and Dentistry, ITTC Building, Tamar Science Park, Plymouth, PL6 8BX UK; University of Exeter Medical School, Peninsula Technology Assessment Group, Veysey Building, Exeter, EX2 4SG UK; BMJ, BMA House, Tavistock Square, WC1H 9JR London

## Abstract

**Background:**

Action taken to enhance or conserve outdoor environments may benefit health and wellbeing through the process of participation but also through improving the environment. There is interest, amongst both health and environmental organisations, in using such activities as health promotion interventions.

The objective of this systematic review was to investigate the health and wellbeing impacts of participation in environmental enhancement and conservation activities and to understand how these activities may be beneficial, to whom and in what circumstances or contexts.

**Methods:**

A theory-led mixed-method systematic review was used to assess evidence of effect and to identify pathways to change (protocol: http://onlinelibrary.wiley.com/doi/10.1002/14651858.CD010351/full). Due to the multi-disciplinary, dispersed and disparate body of evidence an extensive multi-stage search strategy was devised and undertaken. Twenty-seven databases and multiple sources of grey literature were searched and over 200 relevant organisations were contacted. The heterogenous evidence was synthesised using a narrative approach and a conceptual model was developed to illustrate the mechanisms of effect. Due to the limited nature of the evidence additional higher order evidence was sought to assess the plausibility of the proposed mechanisms of effect through which health and wellbeing may accrue.

**Results:**

The majority of the quantitative evidence (13 studies; all poor quality and lower-order study designs) was inconclusive, though a small number of positive and negative associations were observed. The qualitative evidence (13 studies; 10 poor quality, 3 good) indicated that the activities were perceived to have value to health and wellbeing through a number of key mechanisms; including exposure to natural environments, achievement, enjoyment and social contact. Additional high level evidence indicated that these pathways were plausible.

**Conclusions:**

Despite interest in the use of environmental enhancement activities as a health intervention there is currently little direct evidence of effect, this is primarily due to a lack of robust study designs. Further rigorous research is needed to understand the potential of the activities to benefit health and environment.

**Electronic supplementary material:**

The online version of this article (doi:10.1186/s12889-015-2214-3) contains supplementary material, which is available to authorized users.

## Background

There is growing interest in how the environments in which we live, work and play may be supportive of good physical and mental health and wellbeing. Research has demonstrated multiple links between the environment and human health and wellbeing; from the health outcomes which have been shown to be related to proximity and quantity of greenspace in the living environment, to the greater beneficial impact of exercising in the outdoors when compared to indoor settings [1, 2]. Ecological quality is a fundamental factor, with some evidence of variation in health outcome according to exposure to high or low quality environments [3]. Research has also established that the state of the environment is related to health, with, for instance, correlations between environmental incivilities (e.g. litter or graffiti) and reduced wellbeing [4, 5].

Recognition of the potential for these links has led to increasing awareness of potential for the outdoor environment, whether built or natural, to be used as a setting for health-promoting activity [6, 7]. One approach to achieving health-gain though the use of outdoor environments are environmental enhancement and conservation activities, which could be of benefit through the process of active participation but also through improved environments. Such programmes may help address many of the growing health challenges including increasing rates of chronic and non-communicable diseases and the rising costs of health care [8].

Health and environmental organizations in the United Kingdom (UK), United States of America (USA), Australia and elsewhere use such conservation programmes to promote better environments through collective action [see, for example: 9–11]. Improving the health and wellbeing of those participating is central to many programmes, however others are run predominantly to improve the environment with impacts to health and wellbeing incidental. Whatever the specific aims of the programmes there is increasing interest in the approach as a means to improve human health and wellbeing. Claims have been made regarding the potential of these programmes to improve health outcomes such as obesity and mental wellbeing [12, 13].

This paper reports results of an extended systematic review (the protocol for the initial review was registered with the Cochrane Collaboration [14], it should be noted that this review considers participants of any age, whereas the Cochrane protocol and review relate only to adults >18 years). The objective of this review was to deepen understanding as to how participation in environmental enhancement and conservation activities may benefit health or wellbeing. Using a mixed-method theory-led review, which has methodological and theoretical similarities to ‘realist’ and ‘integrative’ reviews and draws on diverse types of evidence, an understanding of the effectiveness of the intervention was situated within the theoretical, geographic, temporal and socio-cultural context [15–18]. The evidence was brought together in such a way as to provide an exploration and illustration of how environmental enhancement and conservation activities may have a beneficial effect, to whom and in what circumstances or contexts. A conceptual model, derived from the primary evidence was further enhanced by integrating high level supplementary evidence to facilitate an understanding of the complexity of the activities and their *potential* impacts by situating the research against formal and everyday theories of effect [19, 20].

‘Environmental enhancement or conservation activities’ are defined as those in which participation (either voluntary or non-voluntary, such as Community Payback (community service) activities, but not through paid employment) is intended to improve the outdoor environment (either urban or rural) and in which active physical participation is required [21]. Typical environmental enhancement and conservation activities include actions such as habitat restoration, litter picking or the re-greening of urban waste sites. The activities considered for this review are typically, though not necessarily, undertaken in groups and could take place anywhere. Domestic gardening, environmental monitoring and specific care and therapeutic gardening projects are not considered to be environmental enhancement or conservation for the purposes of this review (see [14] for further detail).

## Methods

The review followed a multi-stage process. First, a formal mixed-method systematic review was conducted. Effectiveness was assessed through a synthesis of quantitative studies which had been conducted using the most robust and reliable designs available [22]. Qualitative research, which had been conducted using recognised qualitative methods of data collection and analysis, was sought to assess perceived benefits and mechanisms of effect [23, 24]. Individual studies were considered includable in the review if they were reports of primary research (published or ‘grey’) relating to:

- any *population* of participants (whether voluntary or compelled) of any age (the review reported here includes children and young people, populations which were excluded from the Cochrane review [14]);
- outdoor, physically active environmental enhancement or conservation *activities*;
- activities that occurred in any urban or rural *context* whether built or natural;
- any relevant health and wellbeing *outcomes* whether physiological, physical, mental (including emotional and quality of life), or social (see [14] for example measures of each outcome). Mechanisms known to be determinants of health (i.e. physical activity behaviours) were also considered; and
- (for controlled or comparative designs) any suitable *comparator*.

Formal searches were led by an Information Specialist (CC) and were completed in October 2012. To ensure comprehensiveness evidence was identified using standard formal search methods but from broader contexts than is typical for systematic review [22]. In addition to traditional electronic bibliographic searches (27 databases) and citation chasing, evidence was identified through extensive web- and grey-literature searches and through expert and practitioner consultation (200+ environmental and health organizations: see Additional file 1: Table S1). Searches were limited to research conducted after 1990, reported in English and produced in an Organisation for Economic Co-operation and Development country (to ensure some comparability in context). Search terms related to the activities but not outcomes and were clustered in groups relating to and combining: 1) the act of conservation or environmental enhancement; 2) participatory terms (e.g. volunteering or stewardship); and 3) environmental terms (Additional file 1: Table S2).

KH and RL independently screened titles and, if available, the abstracts of all studies. The full text of studies which met the inclusion criteria were obtained and independently screened by KH and RL. The opinion of a third party (RG) was sought in the event of disagreement. Relevant data were extracted from each study into a tailored extraction form (see Additional file 1: Table S3 for variables for which data were extracted) by KH and RL; each extraction was assessed for accuracy by the second reviewer (KH or RL). The PROGRESS-Plus framework was used to consider equality factors [25]. Study quality and risk of bias were assessed using published criteria: the Effective Public Health Practice Project tool for quantitative evidence [26] and the Wallace criteria [27] for qualitative.

Due to the heterogeneity of the evidence identified, a ‘narrative’ approach to synthesis was deemed most appropriate. Narrative synthesis uses words and text rather than solely relying on statistical meta-analysis [28]. Initially the quantitative and qualitative data were summarised and synthesized separately. The quantitative evidence was found to be heterogeneous in design, measures used/applied and populations studied; therefore it was not possible to use formal meta-analysis to establish pooled effect measures. Instead summaries of effectiveness according to design, measures and theory were produced [28]. The qualitative data were summarized and grouped according to key themes and concepts with quotes used to illustrate findings [29]. Although the quantitative evidence was interrogated for sub-group variation, according to factors considered to be of potential interest: a) those reporting participants with mental ill-health; b) programme type; and c) the specific quality of life assessment tool used, the heterogeneity in evaluative approach and paucity of detail regarding the interventions and participants meant that formal sub-group analyses were not appropriate. The separate quantitative and qualitative syntheses were brought together for the overarching analysis.

The second stage of the review constituted the creation of a conceptual model of effect [19, 20, 28]. The model version reported here developed through a series of iterations that were referred to the two expert advisory groups for comment. The model was informed by the initial quantitative review results and then expanded through further analysis of the qualitative data. The conceptual model illustrates the processes, moderators, and mediators relating to the *potential* health and wellbeing impacts of environmental enhancement and conservation activities.

The third stage of the review related to the use of supplementary evidence which was sought to assess whether the specific process outcomes and intermediary mechanisms, which appeared to link the activities to the health and wellbeing benefits observed, were credible. It has been argued that in reviews of public health interventions where it is likely there is only a small body of directly relevant research it is beneficial and legitimate to use additional evidence to further understand the issues [23, 30]. Four key processes common to many of the studies and through which it was plausible that positive impacts may have come about were prioritised and recent evidence from higher order study designs relating to relevant populations identified [31]. The supplementary searches for high–level evidence relating to the four key mechanisms of change were carried out by RL and CC, and experts relating to each theme were consulted as to further sources of evidence.

An Expert Advisory group comprised of methodological experts provided advice on the review techniques and a Project Reference group, comprised of those funding, supporting or delivering the activities, advised on matters relating to the environmental enhancement and conservation activities.

## Results

### The evidence

The primary bibliographic searches identified a total of 16,573 unique references; 16,463 of these were excluded at title and abstract (see Additional file 1: Figure S1 for results flow diagram). A further 211 studies were identified through the searches of grey literature and following contact with relevant organisations. Three hundred and twenty one full text articles were retrieved and assessed against the inclusion criteria, 289 of which were excluded (mostly for not detailing empirical research; detailing activities or location of activities not meeting our criteria of environmental enhancement and conservation activities; and for methodological reasons).

Thirty-two papers detailing research relating to 23 individual interventions (10 quantitative, 10 qualitative and 3 mixed method) were included in the synthesis, multiple papers related to individual interventions (see Table 1 for groupings).

**Table 1:** Study Characteristics

Primary ref	Ref	Additional paper refs	Study type and report type	Country	Design	N	% women	Mean age	Participants	Activity descriptors and environment	Intensity	Comparator	Quality grading
Barton et al. 2009	[46]	[91, 92]	Quantitative, thesis and peer reviewed articles	UK	uBA	19	26	~60	Volunteers	Conservation. Rural natural environment.	NR	Active recreation	Weak
Brooker and Brooker 2008^a^	[42]^a^		Quantitative, unpublished case study	UK	N of 1 uBA	1	100	49	Volunteers	Green Gym. Rural natural environment.	“Regular”	Other physical activity	Weak
Brooker and Brooker 2008a^a^	[43]^a^		Quantitative, unpublished case study	UK	N of 1 uBA	1	100	49	Volunteers	Green Gym. Rural natural environment.	“Regular”	Other physical activity	Weak
BTCV 2009	[36]		Quantitative, unpublished evaluation report	UK	uBA	122	47	9	School pupils	Green Gym. Mixed natural environments.	1 – 1.5 hours per week	N/A	Weak
Eastaugh et al. 2010	[33]		Quantitative, unpublished evaluation report	UK	uBA	8	NR	NR	Referred and voluntary	Conservation. Woodlands.	NR	N/A	Weak
Moore et al. 2006	[44]		Quantitative, peer reviewed article	Australia	Case control	102	37	55	Volunteers	Conservation. Rural natural environment.	NR	Community members not involved in conservation	Weak
Pillemer et al. 2010	[45]		Quantitative, peer reviewed article	Canada	Cohort	2630	57	45	NR	Conservation. No detail on environment.	NR	Alternative volunteering	Weak
Reynolds 1999a	[47]	[93]	Quantitative, peer reviewed article and unpublished evaluation report	UK	uBA	16	49	60	Volunteers	Green Gym. Rural natural environment.	3 hours twice a week	N/A	Weak
Small Woods 2011	[40]	[94]	Quantitative, unpublished evaluation reports	UK	uBA	7	100	NR	Referred	Woodland management. Woodlands.	NR	N/A	Weak
Yerrell 2008	[41]		Quantitative, unpublished evaluation report	UK	uBA	194	40	45	Volunteers and referred	Green Gym. Mixed natural environments.	1-4 hours once a week	N/A	Weak
Birch 2005	[48]		Qualitative, peer reviewed article	UK	Interviews, ethnographic	3	67	42	Volunteers	Green Gym. Rural natural environment.	3 hrs twice weekly	N/A	Poor
Burls 2007	[34]		Qualitative, peer reviewed article	UK	Focus groups, ethnographic	11	NR	NR	Volunteers	Conservation gardening. Urban natural environments.	NR	N/A	Poor
Carter and O'Brien 2008	[39]	[95]	Qualitative, unpublished evaluation report	UK	“qualitative evaluation”	NR	NR	NR	Referred	Habitat Restoration. Rural natural environments, woodlands	1-2 days per week for 6 months	N/A	Poor
Christie 2004	[49]		Qualitative, unpublished report	Australia	Interviews	18	NR	30	Volunteers	Bush Regeneration. Rural natural environment.	NR	N/A	Poor
Gooch 2005	[35]		Qualitative, peer reviewed article	Australia	Interviews	85	NR	NR	Volunteers	Conservation activities. Natural environments, water catchments.	NR	N/A	Poor
Halpenny and Caissie 2003	[50]	[96]	Qualitative, peer reviewed articles	Canada	Interviews	10	50	40	Volunteers	Restoring habitats. Rural natural environments.	3-17 day vacations	N/A	Good
Miller et al. 2002	[37]		Qualitative, peer reviewed article	USA	Focus groups, ethnographic	30	NR	19	Students	Trail creation. Urban natural environments.	1 day per week for 10 weeks	N/A	Poor
O'Brien et al. 2011	[53]		Qualitative, peer reviewed article	UK	Ethnographic	10	40	40	Volunteers	Wildlife gardening. Urban natural environment.	2-3 days per week	N/A	Good
Townsend and Marsh 2004	[52]		Qualitative, unpublished evaluation report	Australia	Interviews, focus groups	NR 18+	33	65	Volunteers	Restoration of reserve. Rural natural environment.	NR	N/A	Poor
Townsend 2006	[51]	[97]	Qualitative, peer reviewed article and unpublished evaluation report	Australia	Interviews	35	NR	NR	Volunteers	Maintenance of creek and reserves. Rural natural environment.	NR	N/A	Poor
BTCV 2010	[38]		Mixed, unpublished evaluation report	UK	uBA	136	NR	NR	Volunteers and referred	Green Gym. Rural natural environment.	NR	N/A	Weak
					Interviews	19	16	NR					Poor
O'Brien et al. 2010	[54]	[98]	Mixed, peer reviewed article and unpublished evaluation report	UK	uBA	88	28	43	Volunteers	Vegetation Clearing. Rural natural environment.	0 - 33 hrs per month	N/A	Weak
					Interviews, ethnographic								Good
Wilson 2009	[32]		Mixed, unpublished evaluation report	UK	uBA	77	26	41	Referred	Conservation. Rural natural environment, woodlands.	3 hours per week for 12 weeks	N/A	Weak
					Interviews								Poor

^*a*^Studies not included in the synthesis due to small n. uBA, uncontrolled Before and After study. N/A, Not Applicable. NR, Not Reported

### The environmental enhancement and conservation activities

The majority of the evidence (16 of the 23 studies) concerned UK based environmental enhancement and conservation activities. The rest of the evidence related to Australia, Canada or the USA (Table 1). The evaluations predominantly focused on group based activities intended to improve, conserve or develop the outdoor natural environment. The quality of reporting made overall assessment and classification difficult, but the types of activities undertaken by participants included watershed restoration, conservation of natural urban areas, woodland management and path creation. There was generally little description of the specific environment types the activities were undertaken in, however they included woodlands [32, 33], urban wildlife gardens [34], and water catchments [35].

Only one study provided a detailed description of the specific activities undertaken by each participant [32], therefore it is unclear in the majority of the studies how long the environmental enhancement sessions lasted, what specific activities were undertaken, nor of their intensity and frequency.

### Study participants

Participants were predominantly adult, with an average age of approximately 40–60; just two studies reported evaluations of programmes working with children or young people [36, 37]. There was very little detail regarding the socio-economic or educational status of the participants. Participation in the majority of the programmes appeared to be voluntary, where it is assumed people take part for enjoyment, leisure, or environmental and community concerns. Some evaluators described participants being referred to the programme through mental health and social services or probationary programmes [32–34, 38–41].

### Study designs and methodologies

Ten of the studies used quantitative designs to evaluate the impacts of the activities: three case control studies [42–44]; one retrospective cohort study [45]; and six uncontrolled before and after studies [33, 36, 40, 41, 46, 47]. Ten studies used qualitative designs [34, 35, 37, 39, 48–53]. The final 3 studies used mixed methodologies of uncontrolled before and after studies with qualitative components [38, 41, 54]. Recruitment to the studies was not adequately described in any of the papers and there is a risk of potential selection and retention bias. Further detail regarding sample size, basic demographics and comparator group/activity can be found in Table 1.

### Assessments of study quality

The quality of all included quantitative studies was poor (Additional file 1: Table S5). Issues related to weak study designs (including a longitudinal cohort study [45]), serious potential of multiple forms of bias, and inadequate reporting. In just under half of the quantitative studies the sample sizes were small (<n20) [33, 40, 46, 47] compromising the validity of any statistical analyses carried out (reporting of these small n studies are highlighted in the results section with a † symbol). The reliability of two studies [42, 43] was deemed too low for the evidence to be included in the synthesis; this was due to the use of an ‘n of 1’ case study research design. Only five of the quantitative studies had been published in peer reviewed journals [44–47, 54].

Three of the qualitative studies were rated as ‘good’ [50, 53, 54], the rest were ‘poor’ quality (Additional file 1: Table S5). There was insufficient detail regarding activities and participant characteristics in the majority of the qualitative studies. Eight studies were unpublished (within the academic literature) programme evaluations which had, therefore, not been subject to formal peer review [32, 33, 36, 38, 40–43, 52].

### Quantitative outcome measures

The majority of the studies used self-report measures to assess impacts on health or wellbeing outcomes, with a small number using objective tools such as blood pressure monitors [41–43]. Three studies considered self-reported physical activity rates; only one of which used a validated scale, the Scottish Physical Activity Scale [32], the other two used bespoke scales [36, 45]. Mental health outcomes were assessed in five of the included studies; measures included the Warwick and Edinburgh Mental Wellbeing Scale [32], Rosenberg Self-Esteem Scale and Profile of Mood States scale [46], and the Emotional States Scale [54]. The other two studies to consider mental health outcomes used measures devised specifically for the study [44, 45]. Quality of life (QoL) was assessed using measures such as the Short Form family (e.g. SF-36 or −12), used in six of the evaluations [32, 33, 38, 40, 41, 47], the Paediatric QoL inventory [36] and other bespoke measures [44, 45]. One study assessed social function using the Bruckner Community Cohesion scale [44]. Three studies objectively assessed physiological outcomes including heart rate, aerobic capacity and blood pressure [41–43].

### Qualitative themes

The qualitative studies, most of which used semi-structured interviews or focus group methodologies and a thematic approach to analysis, sought to understand the links between participation in environmental enhancement activities and health and wellbeing outcomes by exploring a number of key over-arching themes (Table 2).

**Table 2:** Qualitative findings by main overarching theme

Ref	Ref	N.	Method	Quality	Personal achievement	Personal/ social identify	Developing knowledge	Benefits of place	Social contact	Physical benefits	Physical activity	Spirituality	Psychological restoration	Risks/ negatives
Birch 2005	[48]	3	Interviews	Poor	X			X	X		X		X	
BTCV 2010	[38]	19	Interviews	Poor	X		X	X	X	X		X	X	X
					X		X	X	X				X	X
Burls 2007	[34]	11	Focus Groups	Poor	X	X	X	X	X	X	X	X	X	
Carter and O'Brien 2008	[39]	NR	"Qualitative evaluation"	Poor	X	X	X	X	X		X		X	
Christie 2004	[49]	18	Interviews	Poor	X	X	X	X	X			X	X	X
Gooch 2005	[35]	85	Interviews	Poor	X	X	X	X	X				X	X
Halpenny and Caissie 2003	[50]	10	Interviews	Good	X			X	X				X	
Miller et al. 2002	[37]	30	Focus Groups, ethnographic	Poor	X		X	X	X				X	X
O'Brien et al. 2010	[54]	88	Interviews	Good	X		X	X	X	X		X	X	
O'Brien et al. 2011	[53]	10	Ethnographic	Good	X	X	X	X	X	X	X	X	X	
Townsend and Marsh 2004	[52]	18	Interviews	Poor	X		X		X	X	X		X	
					X		X	X	X	X	X		X	
Townsend 2006	[51]	35	Interviews	Poor	X			X	X		X		X	
Wilson 2009	[32]	29	Interviews	Poor	X	X	X	X	X				X	X

### Quantified impacts of environmental enhancement and conservation activities to health and wellbeing outcomes

The synthesis of the quantitative outcomes omits the results of two studies [42, 43] because of small participant numbers (both are n of 1 case studies). The study design was not considered to be robust enough for inclusion.

There was little quantitative evidence of positive health and wellbeing benefits from participating in environmental enhancement and conservation activities. All studies were found to be of poor quality and subject to several potential sources of bias. Most outcomes reported in quantitative studies were not statistically significant, drawn from small samples (<20 meaning that statistical analyses are problematic, highlighted below with a †), or were inconsistent. There was however some very tentative evidence to suggest that participation in environmental enhancement and conservation activities could have a beneficial impact to the primary health and wellbeing outcomes or intermediary factors considered in this review (Table 3), there were also a small number of negative outcomes.

**Table 3:** Quantitative findings

Ref	Ref	N.	Method	Quality	Physiological	Physical	Mental and emotional	Quality of life	Social/other
BTCV 2009	[36]	122	uBA	Weak		↑		↑ (↑ →)	
BTCV 2010b	[38]	136	uBA	Weak				(→→)	
Eastaugh et al. 2010	[33]	8	uBA	Weak				→(→→→)	
Moore et al. 2006	[44]^a^	102	Case–control	Weak			↑↓→→	↑↑↑→→→→	↑↑↑↑↑→→→→→→→→→→→→→→
O'Brien et al. 2010	[54]	88	uBA	Weak			↑		
Pillemer et al. 2010	[45]^a^	2630	Cohort	Weak		↑↑	↑ →	↑	
Barton et al. 2009	[46]	19	uBA	Weak			→→		
Reynolds 1999a	[47]	16	uBA	Weak	↑→→→→→→→→			(↑↑→→→→→→)	
Small Woods 2011	[40]	7	uBA	Weak				→→(→→→→→→)	
Wilson 2009	[32]	77	uBA	Weak		↑	→	→ → (→→ →→→→→→)	
Yerrell 2008	[41]	194	uBA	Weak				↑↓	

^a^Comparative design. () Domains of the SF-36/12. ↑ Significant positive change.

→ No significant difference. ↓ Significant negative change. Each individual arrow refers to an individual outcome as detailed in the study (e.g. study 47 included 9 different physiological measures, 1 of which was positive and 8 inconclusive).

In the one study to have assessed physiological outcomes a positive change was observed in grip strength, but not in aerobic capacity, BMI, weight, body composition, flexibility, blood pressure, balance or hip/waist ratio) [47].

No primary physical health measures were used in any of the included studies. Physical activity (as measured using the Scottish Physical Activity Questionnaire and self-reported levels) was found to have increased following participation in the two studies that reported assessing this outcome [32, 36]. An association between conservation volunteering and physical activity was observed in the retrospective cohort study [45].

Whilst the majority of the measures of positive mental health and wellbeing states were inconclusive there were some significant associations reported in three of the five studies to assess this outcome [44, 45, 54], and one study [44] reported a negative effect on mental health.

The majority of the quality of life measures were inconclusive, however five studies found some positive associations with participation. Quality of life benefits measured using SF36/12 were observed for both adults [41, 44] and children [36]. There was an association between conservation volunteering and quality of life in the cohort study [45]. In the one study which considered social function a positive association was reported [44]. One study found a negative association between participation and quality of life [41].

Despite some tentative links the majority of the evidence was drawn from poor quality evidence, inconclusive, and with variation in the direction of particular outcomes within and between studies.

#### Subgroup variation

It was not possible to reliably assess variation in impact according to activity type, environment or according to any of the usual demographic factors (such as age, employment etc.) or according to the following three groupings that were anticipated to be of interest:

1. Participants referred to the programmes by health or social services [32, 33†, 38†] as opposed to more ‘traditional’ voluntary participants.
2. Participants with mental ill health [33†, 40†, 41] against those where no such condition was reported.
3. Formal ‘branded’ programmes such as the Green Gym [10, 46†, 47†, 41, 48] against other informal types of programmes and activities. Green Gyms are programmes typically run by environmental organisations and are intended to provide an alternative to the indoor gym environment and provide physical activity through conservation work.

This was due to the poor quality of the studies and the inadequate and inconsistent reporting.

### Qualitative findings

The included qualitative studies were used to expand on the findings of the quantitative synthesis to explore the ways in which the activities were experienced. Whilst three studies were found to be of good quality [50, 53, 54] the rest were poor (see Additional file 1: Table S5), meaning the results taken from those studies should be treated with caution. Further the research participants appeared to be a predominantly self-selected group meaning that they may have had more favourable perceptions of the programmes and may have anticipated beneficial experiences. However it should be noted that each of the main qualitative themes (apart from the theme of ‘risk’) were present in one or more of the three studies rated as being of ‘good’ quality (see table 3).

The majority of the participants perceived that their health and wellbeing, whether physical or mental, improved following engagement in the activities. The discussion within the qualitative evidence of wider factors associated with the programmes and participants’ response is also suggestive of the pathways through which the benefits are achieved and accrued. Many of these pathways can be conceived of as mechanisms of change or as independent impacts in their own right and are, therefore, discussed here.

#### Direct impacts to physiological and mental health and wellbeing

There was relatively little discussion of ‘actual’ or perceived physical health change within the studies using qualitative methodologies, however what was reported was positive. Eight of the mental-health service clients in the Scottish ‘Branching Out’ programme did feel that their health had improved:

*“I feel it’s actually benefited my health, because I do suffer from asthma. It seems as if I’m getting more fresh air and I feel a wee bit healthier and plus some of the work that they dae, I feel that, in a way it is making me lose a wee bit of weight. I used to be twenty stone now I’m only eighteen”* [Participant. 32]

More common was discussion of greater physical fitness attributed to participation in the programmes, this was achieved either through physically demanding activities [52, 54] or through reduced opportunities for damaging behaviours: *“…keeps my mind occupied and off the booze for a few hours”* [Female participant. 38].

The impacts, and potential pathways, to better mental health and wellbeing were discussed in all studies. Specific outcomes included feeling calmer [34], lowered stress levels [38], more positive outlook [39], greater feelings of self-worth [35], and enhanced resilience and capacity to manage life events and illness [34, 38, 48, 53]. For many participants the enhancement activities were psychologically therapeutic [32, 34, 38, 48, 50–52, 54]. Of particular importance was the promotion of a state of tranquillity:

*“Just even, like peace of mind as well. There’s something about being outdoors that I think just gives you calmness”* [Participant. 50]

Mechanisms linking the activities to better mental health included: gaining pleasure and happiness from participation [35]; the mental stimulation of complex tasks [48, 54]; the relaxed atmosphere of the groups [54]; enhanced confidence, assertiveness and sociability [38]; and the social contact and support [32, 37, 39, 51–53].

#### Achievement and sense of reward from activities

Many of the studies using qualitative methodologies explored the notion that achievement, contribution to the environment and society, and personal satisfaction through rewarding activities, could act as pathways through which the benefits of participating in environmental enhancement and conservation could be realised.

Involvement was linked to altruistic feelings which were in turn associated with wellbeing; participants spoke of activities providing an opportunity to repay ‘debts’, to give something back to an environment, community or society [34, 49, 50, 52, 54]. A UK conservation volunteer who was recovering from mental ill-health stated:

*“I am part of a group, a city, a country and a society which can take care of its vulnerable members, of which I was one, and this has benefited me greatly and I feel I want to give something back to this culture and to nature in general by coming here and making an effort”* [53].

Fostering a sense of achievement through participation was of particular importance and the actual goal or outcome achieved could take many forms, from small life-style changes through to considerable societal contributions. For some, particularly those suffering from mental ill-health, participation provided the motivation and a structure through which to involve themselves in their community [32, 38, 51]. For these participants, the programmes were therapeutic, much of which was attributed to the achievement of small goals, even just being able to leave the house. Participation was described, by one Australian volunteer, as *“like a dose of medicine”* [51]. For others achievement was felt through making a tangible impact on a degraded landscape [35, 48, 50, 52]. The benefits of achievement were linked to the perception that the activities, and the results of their efforts, were rewarding and a positive use of time and energy:

*“…I wasn’t too good at it [willow weaving] but at the end I done it. At least I tried …I feel in myself I’ve achieved something …Like see when I gae home after leaving here I’m puffed oot and I feel as if I’ve achieved something. I’m knackered and I’m quite proud of myself cause I’ve done it.”* [Participant. 32]

Christie [49] linked the potential psychological benefits to the nature of the conservation and enhancement activities; suggesting that the tangible and physical change which occurs, the empowerment of affecting these improvements and the small individual contributions summing to a larger group achievement provide a link between activity and impact. Miller et al. [37] suggested that through the process of participation a sense of pride in place, of ‘ownership’ developed, further promoting wellbeing:

*“One participant brought the other group of home residents and staff out to the trail to show off what he had accomplished… "It's their trail, you can see the pride and ownership; it's theirs. It's something they worked on, something that they value”.”* [Author and programme leader. 37]

Taking action to protect the environment was also an important factor in motivation to take part for the participants in six of the studies [34, 38, 48–50, 53]. The multiple beneficiaries of the environmental improvements (personal, environmental, social and cultural) conferred a sense of satisfaction, as did the perception that they, though small local actions, were contributing to a larger movement. This was particularly evident for those volunteers working on smaller scale environmental improvements and where there was visual impact [49]. However there were indications that a sense of obligation or even of ‘well informed futility’ could develop and this, for some, led to negative psychological impacts [50]. Gooch [35], who focused on Landcare volunteers in Australia, also found less positive responses amongst volunteers who worked to conserve the ecosystems and landscape features integral to local economies and cultures:

*“There’s a need here, I don’t enjoy this [volunteering] at the moment, I must admit it, it’s… it’s killing me, but I’ve got to keep going, there’s just too much at stake”* [35].

This participant voiced the distress he felt which drove him to continue, but illustrates that the drive to conserve the natural environment can be powerful motivator.

#### Skills and learning

Opportunities to learn new skills were linked to wellbeing benefits by the authors of a number of the studies included in the review [32, 34, 35, 37, 38, 52, 54]. Increases in self-esteem and confidence through the acquisition and sharing of knowledge were noted [38], as was the potential increase in employability [54]. For others participation promoted a sense of self-efficacy, which facilitated and supported further action, for example in local government [52].

#### Benefits of the environment/context

In the majority of the studies a link was made between the spaces in which the programmes took place and the perceived health and wellbeing benefits. For some, contact with and activity in the natural environment was of particular importance [38, 50, 51, 54]. The psychologically restorative and recuperative qualities of natural environments constituted a further pathway between the activities and enhanced wellbeing [34, 38, 48, 50–54]. Burls [34] described the physical environment as a crucial medium in which positive interactions between [mental health] service users and therapist were facilitated; *“Nature doesn’t answer back or judge, it holds no spite or malice”* (Author). In five studies the facility of the natural environment to promote spiritual growth was noted [34, 38, 49, 53, 54]. Being away from stressors was a fundamentally important aspect of the programmes for both traditional conservation volunteers and those who had been referred to the programme through social or health services [32, 34, 48, 50, 51, 53, 54]. The environments in which the programmes took place (these were, as far as could be ascertained, predominantly natural) were described as simple, peaceful, and, crucially, ‘away’ or ‘other’ to usual environments [34, 38, 52, 54]. Burls [34] suggested that one of the primary benefits of the environmental enhancement and conservation programmes for people suffering from mental ill health was that they took place in neutral environments which had significantly calmer and more positive atmospheres than the more traditional setting of the mental health services day centre.

#### Social contact

Each of the studies identified and included in the review focused on group based environmental enhancement and conservation activities, and each study drew links between the social contact gained through participation, and wellbeing.

Participants in a number of the studies were experiencing or were vulnerable to social isolation [32, 37–40, 53, 54]. The retired volunteers who spoke to O'Brien, Townsend and Ebden [54] found value in the widening of their social circles which had naturally narrowed after finishing work. These participants maintained a sense of worth and status, which contributed to their quality of life, through their role as conservation volunteers. For those with depression and other mental ill-health, participation provided a motivation to get out the house and the social support they experience further reinforced their motivation [32, 38, 53]. While it was not a key factor for all [52], the relationships which were formed and which endured beyond the programme [54] were perceived to have promoted enhanced wellbeing. Group based voluntary action in local communities enhanced social cohesion and capital [48] and promoted a sense of belonging [34, 51].

The neutrality of the interaction was important for many;

*“We all get on very well it’s quite a close band of people. There’s no hidden agenda; you don’t need to know who the people are or what they do. You just come [and] enjoy the day that’s the beauty of it.”* [Participant. 54].

This was of particular relevance to those participants who were experiencing mental illness [38, 51], who lived in or spent time in an institution [32, 34] and for the ex-prisoners [39]. These groups reported experiencing reduced stigmatisation and alienation during their participation in the activities; their ‘identity’ was as conservation volunteers rather than as mentally ill patients, or prisoners. For the ex-prisoners the responsibility and trust associated with the role was important; *“It’s nice feeling part of, ehm, part of society again…”* [Participant. 39].

#### Benefits of a structured programme

For some the structure of the programmes was in itself beneficial; evaluators noted that various programme factors, whether they were the rewarding activities, working in the outdoors, or opportunities for supportive social contact, provoked small changes in individuals [38]. These changes included promoting the self-motivation to attend the sessions, which led to bigger changes in employability, social integration and so on [32, 34, 38, 48, 50, 51, 54]. However for others, particularly those who were likely to be volunteering for leisure purposes, the relaxed nature of the activities, the freedom that participants had to work at their own pace, to take on roles which suited them, and to work in an outdoor rural setting, was considered beneficial and contrasted with the ‘stress’ of their everyday urban lives and work patterns [34, 35, 37, 38, 44, 54].

### Conceptual model and supplementary evidence

**Fig. 1:**
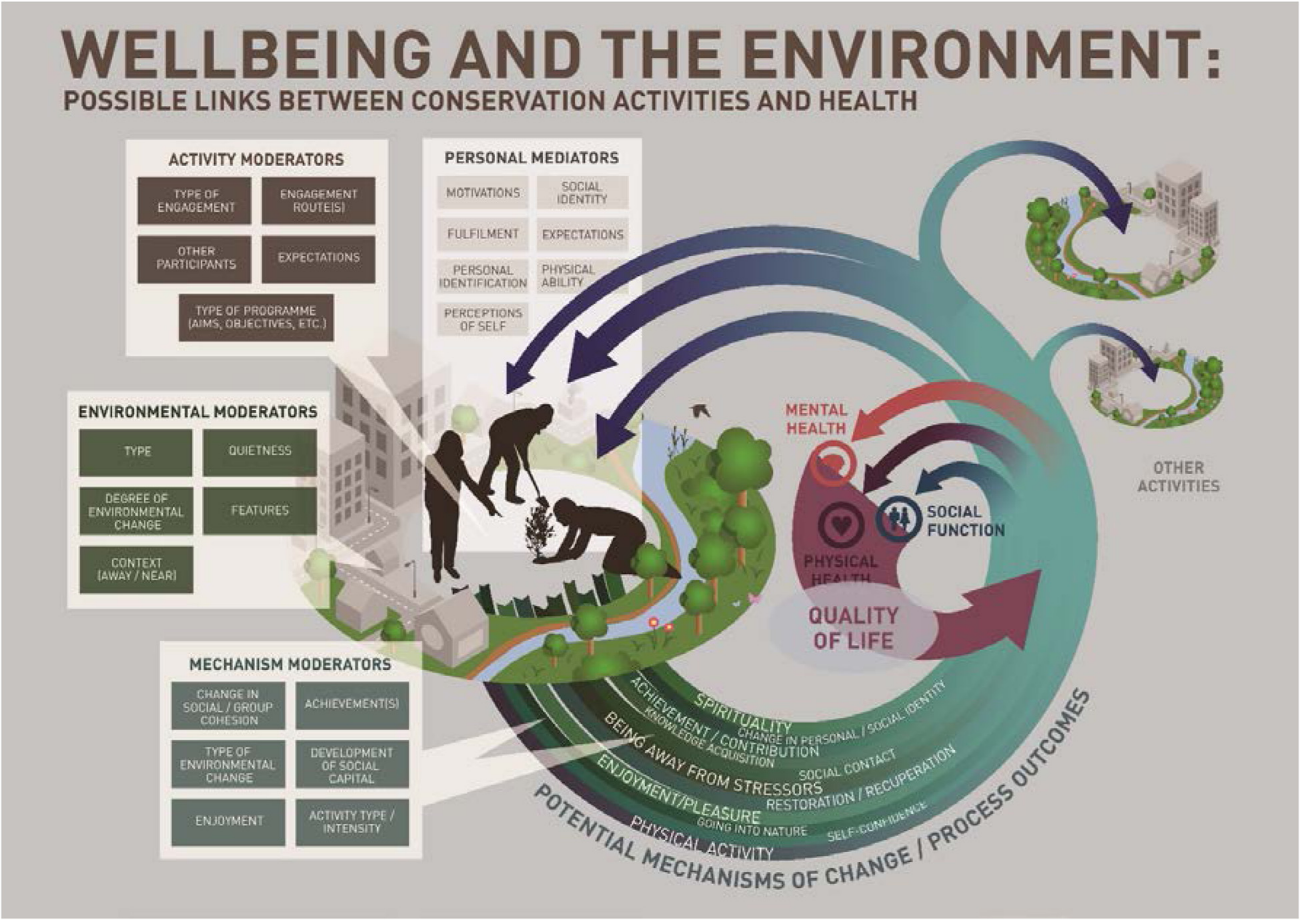
Conceptual model of effect

The conceptual model (Fig. 1) is illustrative of the *potential* pathways to impact. The model was informed by both the quantitative and qualitative syntheses. The outcomes included in the model (mental and physical health, social function, and quality of life) were those that were considered in the quantitative studies. The activities and outcomes are linked in the model by a number of pathways, these were derived from the qualitative synthesis and represent the ways in which perceived health and wellbeing outcomes were considered, by the participants (a predominantly self-selected group) and evaluators, to come about. These pathways are sometimes referred to as ‘everyday’ theories of effect [15].

#### Mechanisms of change and pathways to impact

The environmental enhancement and conservation activities are linked to the potential outcomes by ‘mechanisms of change’. Many of these mechanisms could be considered as process or intermediate outcomes in their own right; for example increased opportunities for acquiring new knowledge and skills may improve final health outcomes such as mental wellbeing. The mechanisms of change and process outcomes were derived from the primary evidence and are broad categories; many have several subthemes not shown on the model. Four of the mechanisms of change - physical activity, achievement and contribution, social contact, and exposure to the natural environment - were of particular importance and may be responsible for many of the positive impacts observed. The supplementary evidence detailed below tests the legitimacy of the four key pathways (greater detail for each piece of supplementary evidence can be found in Additional file 1: Table S6) [31].

#### Pathway 1. Physical activity

Physical activity is likely to be one of the key mechanisms by which environmental enhancement and conservation activities benefit physiological, physical, mental and social health and wellbeing. While the specific benefits depend on the types, frequency and duration of the physical activities, detail which is absent from the majority of the studies, the quantitative and qualitative evidence included in this review are indicative that the link is likely. There is good higher order evidence to suggest that physical activity is beneficial throughout the life course [55, 56]. Systematic reviews of the evidence have established links between adequate levels of physical activity and: adiposity [57–59]; reduced rates of depression and anxiety [60, 61]; reduced incidences of certain cancers [62]; decreases in cardio-vascular and all-cause mortality [63]; and good bone mass, muscle strength, balance and endurance [64, 65]. There is also a body of evidence which indicates that interventions using physical activity are effective at preventing conditions including childhood asthma [66] and cerebrovascular disease [67], in treating conditions such as depression [68], or in the promotion of recuperation from diseases such as cancer [69].

#### Pathway 2. Achievement and contribution

In each of the qualitative studies it was suggested that participation in environmental enhancement and conservation activities could promote wellbeing through achievement or contribution. The origins of the sense of achievement were broad; ranging from reaching personal milestones [35] to overcoming life-limiting psychological states [32]. The perception of making a contribution related to making real and appreciated differences to local environments [49], giving back to a community that had previously been a source of support [53], or through a commitment to a global movement [34]. As illustrated in the model, achievement and contribution were thought to relate to both mental health and social function. The link between these types of achievement and contribution to mental and social health and wellbeing is well researched and evidence suggests it is plausible. German longitudinal research suggested that committed social and political involvement promote greater life satisfaction [70]. This is supported by the UK’s Mental Health Foresight review which concluded that intentional activities, including ‘striving towards goals that reflect deeply-held values rather than being driven by external rewards’, are strongly related to psychological wellbeing [71]. Formal volunteering has repeatedly been shown to be related to good health and wellbeing outcomes [72]. Volunteering activity post retirement has been linked to better self-rated health, functioning, physical activity and life satisfaction as well as to decreased depression and mortality [73–75]. The benefits of achieving personal goals through, for example, lifelong learning is also well evidenced, with links to wellbeing, mental health resilience and recuperative capacity, and cognitive ability [76–78].

#### Pathway 3. Social contact

Several of the environmental enhancement and conservation programmes were run with the intention of facilitating and increasing social contact [32, 38, 47, 51]. This was achieved through several routes; some were direct and involved programme leaders identifying and engaging the participation of socially isolated individuals or groups [32, 38, 51], others were indirect and through voluntary action to improve local communal spaces hoping to facilitate greater community use and subsequent cohesion [34, 49]. Again there is good quality, robust evidence which has demonstrated the health and wellbeing benefits of social contact, reduced social isolation, and of communities with greater social capital. A large scale meta-analysis of 148 prospective studies concluded that stronger social relationships were associated with a 50 % reduction in mortality and that the influence of social relationships on death were comparable to other risk factors such as smoking or alcohol consumption [79]. Social capital also has strong links with mental wellbeing [80]. High levels of social capital (which can be developed through volunteering) are protective of health and quality of life amongst older people through, for example, the provision of informal support [74, 81] and is associated with reduced risk of dementia and Alzheimers [82]. Communities and neighbourhoods with ‘favourable social climates’ are beneficial to the health of children [82].

#### Pathway 3. Contact with the natural environment

Several of the studies make use of well-established theories, for example Attention Restoration [83], Biophilia [84] and Psycho-physiological [85] theories, which suggest that contact with the natural environment may be one way through which participants benefit from involvement in the programmes. The qualitative evidence supported these hypotheses, with the majority of the studies suggesting that the natural environment promoted recuperation, feelings of calmness and tranquillity, and more positive psychological states [32, 36, 41, 44–46, 48, 52]. In the conceptual model ‘going into nature’ was included as a potential mechanism linking the activities to the outcomes and, whilst it is a relatively young field of research, there is some evidence to suggest that this may be a plausible pathway. Meta-analyses have tended to indicate that relationships between use of, or exposure to the natural environment and various health outcomes (including obesity, mental health, response to physical activity, and psychological outcomes such as attention or revitalisation) are positive but weak [1–3, 86–88]. A systematic review of the use of natural settings for therapeutic interventions (initiatives which share similarities to a number of the programmes included in this review [32, 38, 51]), found they were effective, with improvements in conditions such as obesity and schizophrenia [89].

#### Linearity of pathways and impacts

The model illustrates the suggestion that participation in environmental enhancement and conservation activities is likely to be a process subject to variation and feedback loops and, therefore, any outcomes (or processes leading to final outcomes) are neither strictly linear nor independent of each other. For example increased social contact may improve a participant’s confidence which may result in further opportunities for social interaction, ability to take on leadership roles and so on. Evidence for a feedback loop was found in the BTCV’s Wellbeing Comes Naturally programme evaluation [38]; the evaluator highlighted the impacts of greater confidence and sociability gained through volunteering:

*“Respondents felt that volunteers had benefitted over the course of their involvement, with examples including: increased confidence and sociability, becoming less stressed, more relaxed, developing confidence, taking on leading roles. Improved confidence was felt to be linked to enhanced knowledge about how to use tools properly"* [Author].

Further variation in the observed outcomes and in the processes leading to the impacts was derived from the moderating and mediating factors. Moderators were the factors which might have influenced the outcomes or the processes leading to the outcomes. There were three sources of moderators identified in the review evidence: 1) ‘mechanism moderators’ (related to the feedback loops, for instance seeing a change in the environment or gaining enough knowledge through participation to lead sessions); 2) the ‘environment’ in which an activity is undertaken (degraded vs pristine, ‘private’ vs public); and 3) ‘activity moderators’ are those related to the types of activity (high vs low physical impact or the specifics of the programme). ‘Personal mediators’ were included as the evidence suggested that factors such as individual expectations and social identity were important and may have influenced the outcomes. For example Yerrell’s [41] results suggested that those who had had the worst health at entry to the Green Gym programme experienced the greatest improvement. Similarly qualitative evidence indicated that it was the groups experiencing marginalisation, whether through mental ill health or following imprisonment, who spoke of life changing impacts [32, 39, 51]. ‘Motivation’ also emerged as a key factor as to how people approached and potentially benefited from the programme;

*“You’ve got to weigh up your family life on the one hand, and your work and then your… this is just supposedly a recreation, your life is split into thirds, well I don’t have recreation, I have our catchment group. Which is my work, my real work”* [Participant. 35]

Clearly for some, the work done as part of these programmes took on huge significance.

## Discussion

While the majority of the quantitative evidence was inconclusive and drawn from poor quality studies using small scale, lower-order study designs (with predominantly self-selected participants) unsuitable to test relationships robustly, there were tentative indications that environmental enhancement and conservation activities may have some benefit to the health and wellbeing of those participating (however, it should also be noted there was some evidence of a negative impact on aspects of mental health and QoL).

Benefits were thought by those participating in the activities (a mostly self-selected sample) to be achieved through a number of key mechanisms; including time spent in quiet natural environments, achievement, enjoyment and social contact. The qualitative evidence suggested that some of the key pathways, such as social contact and undertaking constructive and valued activities, appeared to be valuable for those (where reporting allowed for assessment) experiencing social isolation and mental ill health. Whilst there was little direct primary evidence within the included studies regarding these pathways and mechanisms, partly due to the lower order quantitative study designs, they were shown to be plausibly linked to health and wellbeing outcomes through the use of the supplementary evidence.

The benefits of the activities perceived by participants in the qualitative research were not reflected in the quantitative evidence. The reasons for this are not clear. It could be that these samples are drawn from different populations, that the benefits identified in the qualitative research (such as increased confidence and social contact) are not measured in the quantitative research or are difficult to quantify, that people perceive benefits that are not in fact at measureable levels, or it may be due to limitations in the study design (such as small sample sizes and short follow up).

The modest indication of some potential positive impacts of the activities are consistent with other related systematic reviews which have suggested that contact with the natural environment is beneficial to health and wellbeing [2] and that exercise in outdoor natural environments is more beneficial than that taking place indoors [1]. A review commissioned by NICE [21] regarding the impacts of changes to environments on physical activity rates (not participative as with this review) found broadly positive outcomes. Furthermore the activities are undertaken to improve the state of the physical living environment and the ‘quality’ of the living environment, whether built [4] or natural [3], has been shown to relate to health outcomes.

Whilst the majority of the primary evidence (both quantitative and qualitative) was of poor quality, thus limiting the synthesis, the reliability of the general findings of the present review were strengthened through the use of relatively novel methodologies including the integration of qualitative evidence and the creation of a conceptual model. Furthermore the conceptual model of potential pathways to impact was strengthened by the use of additional evidence which assessed the plausibility of the mechanisms through which health and wellbeing benefit could be accrued, but which was located outside the original evidence base.

Despite the positive indications there isn’t currently enough robust evidence to allow for definitive and reliable conclusions as to the benefits of environmental enhancement activities (in isolation from other influential factors) to health and wellbeing. There are multiple reasons for this lack of certainty:

- The study designs of the included research were insufficient to show any causal relationships between the activities and outcomes. This was particularly problematic for the studies which focused on marginalised groups, where the activities tended to be part of multi-faceted programmes and were likely to be delivered in conjunction to other interventions (e.g. mental health support). Additionally, and more generally, the outcome measures used in the studies, the majority of which relied on self-report, were not necessarily appropriate to detect what was likely to be relatively small and potentially transient changes in health status. These factors may partially account for the inconclusive findings in many of the quantitative studies. Finally the qualitative and quantitative data is drawn from what appears to be a predominantly self-selected group, there is the potential that these participants elected to take part because they enjoy such activities and expected to benefit.
- Further uncertainty was related to the generally poor level of reporting and description within the studies, this was true for both the academic studies as well as those identified from the grey literature. This lack of information renders a fair assessment of potential sources of bias (particularly selection bias) impossible, resulting in the grading of many studies as ‘poor’ quality.
- Meta- and sub-group analyses of the quantitative findings were not carried out because of a lack of comparability between studies. Even when the same assessment tool had been used (for instance the SF36) the method of application and inconsistency in reporting results meant that pooling data was not possible.

Whilst there is uncertainty regarding the actual impacts of environmental enhancement and conservation activities, the small number of positive indications, and generally positive perceptions of the activities by participants do suggest that it would be of value to consider further research. Key areas for investigation include exploring: who benefits from environmental enhancement and conservation activities; in what context are the activities most effective, for instance asking whether those programmes undertaken in natural as opposed to built environments are more beneficial or whether the socio-cultural context is important [2]; which outcomes are most strongly associated with the activities; how environmental outcomes are related to health and wellbeing outcomes; and, finally, how environmental enhancement and conservation activities can be most effectively delivered. These research questions should be investigated using reliable and robust methodologies suitable to the scale, design and aims of the study. In particular there is a need: 1) to use approaches, such as realist and other theory-led methodologies, which are appropriate to address the complexity of the potential impact of the activities [90]; 2) for further, better quality and more extensive qualitative research to deepen our understanding of the experience and acceptability of the activities; and 3) to address the limitations of using self-selecting research participants through adopting suitable sampling and intervention allocation methodologies.

## Conclusions

This review has found that there is little robust quantitative evidence that allows for an assessment of the effect of environmental enhancement and conservation activities on health and wellbeing outcomes. It is not possible, therefore, to conclude whether the continued use of environmental enhancement and conservation activities is justified. Despite this, the qualitative evidence suggested that the activities are valued and are thought, by participants, to contribute to better health and wellbeing. It is plausible that the activities are beneficial as they incorporate factors previously proven to be effective such as physical activity, increased social contact, improved self-esteem through contribution and achievement, and contact with nature. Whilst much of the evidence was inconclusive, the tentative positive indications support a call for further, more robust, research (whether quantitative or qualitative) to understand if the programmes are effective, to whom and in what contexts.

## Additional file

Additional file 1: Table S1. List of organisations and websites searched. An exhaustive list of organisations contacted by the team, as well as websites hand-searched, for relevant studies. **Table S2.** Sample search strategy (MEDLINE) and databases searched. Search strategy submitted to the MEDLINE database is indicative of all database searches, with minor alterations for other databases. **Table S3.** Data extraction forms. Tables showing the variables extracted from both the qualitative and qualitative studies. **Figure S1.** Results flow diagram. PRISMA diagram showing included/excluded number of items at each stage of the review. **Table S5.** Quality/risk of bias assessments. Quality/risk of bias assessments of each study included in the review, as assessed by two team members independently. **Table S6.** Supplementary evidence details. Details of each study included in the supplementary searches. (DOCX 2320 kb)

## Abbreviations

BTCV: British Trust for Conservation Volunteers
NICE: National Institute of Health and Clinical Excellence
QOL: Quality of Life
SF36/12: Short Form 36/12
uBA: Un-controlled Before and After study
UK: United Kingdom
USA: United States of America
