# Supplementary material for: Understanding how environmental enhancement and conservation activities may benefit health and wellbeing: a systematic review
Source: BMC Public Health. 2015 Sep 7;15:864. doi: 10.1186/s12889-015-2214-3 (PMC4561424; doi:10.1186/s12889-015-2214-3)
Supplement: Additional file 1: Table S1. — List of organisations and websites searched. An exhaustive list of organisations contacted by the team, as well as websites hand-searched, for relevant studies. Table S2. Sample search strategy (MEDLINE) and databases searched. Search strategy submitted to the MEDLINE database is indicative of all database searches, with minor alterations for other databases. Table S3. Data extraction forms. Tables showing the variables extracted from both the qualitative and qualitative studies. Figure S1. Results flow diagram. PRISMA diagram showing included/excluded number of items at each stage of the review. Table S5. Quality/risk of bias assessments. Quality/risk of bias assessments of each study included in the review, as assessed by two team members independently. Table S6. Supplementary evidence details. Details of each study included in the supplementary searches. (DOCX 2320 kb) [file 12889_2015_2214_MOESM1_ESM.docx]

**Understanding how environmental enhancement and conservation activities may benefit health and wellbeing: a systematic review**

**Supporting material**

**S1. List of organisations and websites searched (Table S1.**Organisational contacts**)**

**S2. Sample search strategy (MEDLINE) (**Table S2.Search Strategy) and list of databases searched

S3. Data extraction forms (**Table S3. Quantitative data; Table S4. Qualitative data)**

**S4. Results flow diagram (Figure S1. Results flow diagram (PRISMA))**

**S5. Quality assessments (**Table S5. Assessment of bias in quantitative evidence; Table S6. Qualitative study quality)

**S6. Supplementary evidence details (Table S7. Supplementary evidence details)**

**S1. List of organisations and websites searched**

Table S1. Organisational contacts

| 2020 Vision | Hush Farms |
| --- | --- |
| Aaron Pyecroft | Isle of Anglesey County Council |
| Active Wales | Isle of Wight AONB |
| Age UK | Isles of Scilly AONB |
| Ambios | Keep Britain Tidy, Beach Care |
| Arnside and Silverdale AONB | Keep Wales Tidy |
| Avon Wildlife Trust | Kent Downs AONB |
| Bailies of Bennachie | Kent High Weald Partners |
| Basingstoke con vol | Kent nat tr vol |
| BeachCare (Keep Britain Tidy) | LANTRA |
| Berkshire con vol | Lea Bridge con vol |
| B'ham Guild (Broader) | LEAF/Let nature feed your senses |
| Biodiversity SW | Leicester con vol |
| Biosphere CLS | Lincolnshire Wolds AONB |
| Birmingham Guild for Student Volunteers | Liverpool PCT |
| Blackdown Hills AONB | Llyn Peninsula AONB |
| Blackdown Hills Hedge Association | London and w/msex vol |
| Blackwater Valley countryside volunteers | Lothian con vol |
| Bolton conservation vol | Love where you live |
| Bolton Wildlife Programme | Malvern Hills AONB |
| Bournemouth nat tr vol | Manchester nat tr vol |
| Bracknell con vol | Marine Conservation Society |
| British Waterways | Medway Valley Countryside Partnership |
| BVSC (Birmingham) | Mendip Hills AONB |
| Cambridge con vol | MIND (Eco Minds) |
| Camp Kernow | MoD |
| Cannock Chase AONB | Moor Trees |
| Canterbury Environmental Education Center | NAAONB |
| Cardiff con vol | Nat Eng Big Lottery projs |
| Carymoor Env trust | National Parks |
| Causeway Coast and Glens Heritage Trust | National Trust |
| CCD | National Trust for Scotland |
| Change Agents UK | Natural England |
| Chichester Harbour AONB | Natural England |
| Chichester Harbour AONB Officer | Naturally Active project - Kent |
| Chilterns Conservation Board | Neroche |
| City Farms and Community Gardens | New Forest Volunteers |
| Clwydian Range AONB | Newlands Project |
| CN4C | Newquay Zoo |
| CoAST | NHS Forest |
| Coastnet | Nidderdale AONB |
| Community Environmental Trust | Norfolk Coast AONB Partnership |
| Community Payback | Norfolk nat tr vol |
| Confor SW | North Devon AONB |
| Conservation Foundation | North Devon Council |
| Conservation Volunteers Australia | North East Wales Wildlife |
| Cornwall AONB | North Pennines AONB Partnership |
| Cornwall Council | North Wessex Downs AONB |
| Cotswolds Conservation Board | Northumberland Coast AONB |
| Countryside Recreation Network | OPAL |
| Countryside Trust | Outdoor and Experiential Learning |
| Cove Brook Greenway group | Outdoor health forum |
| Coventry nat tr vol | Oxford cons vol |
| CPRE | Oxford Urban Wildlife Group |
| Cranborne Chase and West Wiltshire Downs AONB | Pembroke 21C |
| CRCC | People and Planet |
| CRESH | Plantlife International |
| CSV | Plymouth Environmental Action |
| Cusgarne Organic Farm | Plymouth Student Scientist |
| CVS | PROSPECTS |
| Dartmoor Preservation Association | Quantock Hills AONB |
| Dean Green Team Volunteers | Reforesting Scotland |
| Dedham Vale AONB and Stour Valley Project | Rowhill con vol |
| Derbyshire con vol | Royal Horticultural Society |
| DofE | RSPB |
| Dorset AONB Partnership | Scarborough con vol |
| Durham Uni con vol | Scottish Wildlife Trusts |
| Durlston Volunteers | Scouts |
| Earth Trust | SeaSchool |
| East Devon AONB Partnership | Sheffield W'experience programmes |
| Egham/Staines con vol | Shropshire Hills AONB |
| Environment Kernow | Silvanus Trust |
| Epping forest con vol | Small Woods Association |
| Europarc | SNCV (Sutton) |
| FEVA | SNH |
| Fleet Pond Soc | Snowdonia Society |
| Forest of Bowland AONB | Solway Coast AONB |
| Forest Research | Somerset Community Food |
| Forest School | South Devon AONB Partnership |
| Forestry Commission | South Down National Park |
| Forestry Commission Scotland | South West Environmental Action Trust |
| Forum for Environmental Volunteering Activity | South West Lakes Trust |
| Friends of Par Beach | South West London Environment Network |
| Friends of the earth | Steeple Woodland Reserve |
| Frimley Fuel Allot con team | Suffolk Coast & Heaths AONB Partnership |
| Froglife | Surfers Against Sewage |
| Gibbonsdown and Court Partnership | Surrey Hills AONB |
| Glasgow Con Vol | Sustrans |
| Glentress Trail Fairies | Tamar Valley AONB Partnership |
| Global Boarders | Teignbridge vols |
| Gloucester vale con vol | TFL volunteers |
| Gower AONB | Thames 21 |
| Green Space Community Network | The Conservation Volunteers (BTCV) |
| Green Team | The Mendip Society |
| Greener Ilfracombe | THRIVE |
| Greenham and Crookham con vol | Venture Scotland |
| Greenpeace Cornwall | vInspired |
| Groundwork | Volunteer Bristol |
| Group | Volunteer Cornwall |
| Guernsey con vol | Volunteer development Scotland |
| Haldon Forest Volunteers | Wandle Trust |
| Haldon4Horses | Wednesday con vol |
| Hampshire con vol | West Country Rivers Trust |
| Harlow con vol | Wicken Fen con vol |
| Haven Holidays | Wildlife Trust |
| High Weald AONB JAC | Wirral county vols |
| Highland Environmental Network | Woodland Trust |
| Hill Holt Wood | Wychwood Project |
| Howardian Hills AONB | Wycombe District Council |
|  | Wye Valley AONB |

*Websites*

http://www.dtic.mil/whs/directives/index.html

US Military DoD

http://www.ccw.gov.uk/default.aspx

Countryside Council for Wales

http://www.tsrc.ac.uk/

Third Sector Research

http://www.vssn.org.uk/

Voluntary Sector Studies Network - Journal

http://www.ivr.org.uk/ivr-evidence-bank?q=&t%5B%5D=362

Institute for Volunteer Research

http://www.naturaleconomynorthwest.co.uk/

Natural Economy North West

http://www.oecd-ilibrary.org/;jsessionid=136d54v2tehqa.delta

OECD iLibrary

http://www.oecd.org/department/0,3355,en_2649_33713_1_1_1_1_1,00.html

OECD Environmental Directorate

http://www.epa.gov/

US Environmental Protection Agency

http://hero.epa.gov/index.cfm

Health and Environmental Research Online - US

http://www.eea.europa.eu/

European Environment Agency

http://www.npca.org/

US National Parks Conservation

http://www.environment.gov.au/

Australian Environment Agency

http://www.ec.gc.ca/default.asp?lang=en

Environment Canada

http://www.npws.ie/

Ireland Parks

http://www.epa.ie/

Environmental Protection Ireland

http://www.doeni.gov.uk/niea/

Dept of Env Ireland

http://www.epa.govt.nz/Pages/default.aspx

NZ EPA

http://www.doc.govt.nz/publications/

NZ Conservation Authority

http://www.forestry.gov.uk/publications

Forestry Commission

http://www.forestry.gov.uk/forestresearch

Forest Research

http://www.snh.gov.uk/publications-data-and-research/

Scottish Natural Hertitage

http://www.feva-scotland.org/display/library

FEVA

http://publications.naturalengland.org.uk/

Natural England

http://www.sehn.org/

Science and Environmental Health Network

http://www.sustainweb.org/publications/

Sustain Web

http://www.fph.org.uk/policy%2c_publications_and_events

Faculty of Public Health College - London

http://www.carefarminguk.org/case-studies.aspx

Care Farming UK

http://www.mod.uk/DefenceInternet/MicroSite/DIO/OurPublications/EstateAndSustainableDevelopment/Sanctuary.htm

Sanctuary Magazine, MoD UK

http://www.hphpcentral.com

International 'healthy parks healthy people' network

http://www.ecohealth.net

International association for ecology and health

http://www.ramsar.org/cda/en/ramsar-pubs/main/ramsar/1-30_4000_0__

Healthy wetlands and healthy people initiative of Ramsar Convention on Wetlands

http://www.cbd.int/

Healthy planet healthy people initiative of the convention on biodiversity

www.saveourseine.com/

Save our Seine

http://www.landcareonline.com/; <http://www.landcareonline.com.au/?page_id=9608>

Landcare

**S2. Sample search strategy (MEDLINE) and databases searched**

Table S2.Search Strategy

Database(s): Ovid MEDLINE(R)

Host: OVID

Data Parameters: 1946 to September Week 3 2012

Date Searched: Wednesday October 3^rd^ 2012

Searched By: CC

Strategy Checked by: KH, RL and RG

| **#** | **Searches** | **Results** |
| --- | --- | --- |
| 1 | (conservation$ and natural and environment$ and (renewal or volunteer$ or voluntary or participat$ or practical or regenerat$ or restor$ or maintain$ or care or enhance$ or preserve or creat$ or activ$ or action$ or involve$)).ti,ab. | 377 |
| 2 | (Conservation adj3 interventions).ti,ab. | 29 |
| 3 | ((environmental$ adj3 (conservation$ or volunteer$ or steward$)) and (Regenerat$ or restore or restoration or redevelop or maintain or enhance or preserve or preserving or create or creation or establish or establishing or founding or build$ or cultivat$ or cultivation or participate or participation)).ti,ab. | 73 |
| 4 | (conservation$ adj3 (group$ or volunteer$ or voluntary or association$ or organisation$ or organization$ or participa$ or stakeholder$ or steward$ or trust or ranger$ or activit$)).ti,ab. | 747 |
| 5 | (conservation$ adj5 (nature or rural or countryside or outdoor$ or outside or backcountry or hinterland or outback or wood$ or park$1 or parkland or garden$ or meadow$ or farm$ or (farm adj1 land) or horticultural or floricultural or botanical or arboretum or allotment$ or forest$ or rainforest or moor$ or dale$1 or marsh$ or mountain$ or beach$ or wilderness or landscape$ or tree$ or copse$ or river$ or lake$ or canal$ or waterway or wetland$ or (open adj1 space$) or (protected adj1 area$) or green$ or planning$ or footpath$ or trail$ or coast$ or cliff$ or dune$ or (bio adj1 diversity) or (eco adj1 system) or (protected adj1 area$))).ti,ab. | 1688 |
| 6 | (geoconservation or (geo adj3 conservation)).ti,ab. | 0 |
| 7 | ((activ$ or practical or participat$) adj3 conservation$).ti,ab. | 481 |
| 8 | exp "Conservation of Natural Resources"/ or *Environment/ or *Environment Design/ | 42248 |
| 9 | (volunteer$ or voluntary).ti,ab. or *Voluntary Workers/ or *Consumer Participation/ or *Health Status/ | 199928 |
| 10 | 8 and 9 | 638 |
| 11 | 1 or 2 or 3 or 4 or 5 or 6 or 7 or 10 | 3520 |
| 12 | ((Volunteer$ or voluntary) adj5 (environment$ or nature or rural or countryside or outdoor$ or outside or backcountry or hinterland or outback or wood$ or park$1 or parkland or garden$ or meadow$ or horticultural or floricultural or botanical or arboretum or allotment$ or forest$ or rainforest or moor$ or dale$1 or marsh$ or mountain$ or beach$ or wilderness or landscape$ or tree$ or copse$ or river$ or lake$ or canal$ or waterway or wetland$ or (open adj1 space$) or (protected adj1 area$) or green$ or planning$ or footpath$ or trail$ or coast$ or cliff$ or dune$ or (bio adj1 diversity) or (eco adj1 system) or (protected adj1 area$))).ti,ab. | 1142 |
| 13 | (((voluntary or volunteer$) adj5 (group$ or association or stakeholder$ or steward$ or ranger$)) and (environment$ or nature or rural or countryside or outdoor$ or outside or backcountry or hinterland or outback or wood$ or park$1 or parkland or garden$ or meadow$ or horticultural or floricultural or botanical or arboretum or allotment$ or forest$ or rainforest or moor$ or dale$1 or marsh$ or mountain$ or beach$ or wilderness or landscape$ or tree$ or copse$ or river$ or lake$ or canal$ or waterway or wetland$ or (open adj1 space$) or (protected adj1 area$) or green$ or planning$ or footpath$ or trail$ or coast$ or cliff$ or dune$ or (bio adj1 diversity) or (eco adj1 system) or (protected adj1 area$))).ti,ab. | 667 |
| 14 | *Voluntary Workers/ | 3989 |
| 15 | (environment$ or nature or rural or countryside or outdoor$ or outside or backcountry or hinterland or outback or wood$ or park$1 or parkland or garden$ or meadow$ or horticultural or floricultural or botanical or arboretum or allotment$ or forest$ or rainforest or moor$ or dale$1 or marsh$ or mountain$ or beach$ or wilderness or landscape$ or tree$ or copse$ or river$ or lake$ or canal$ or waterway or wetland$ or (open adj1 space$) or (protected adj1 area$) or green$ or planning$ or footpath$ or trail$ or coast$ or cliff$ or dune$ or (bio adj1 diversity) or (eco adj1 system) or (protected adj1 area$)).ti,ab. | 1253503 |
| 16 | 14 and 15 | 356 |
| 17 | 12 or 13 or 16 | 2010 |
| 18 | (Green$ adj3 (space$ or gym or exercise or volunteer$ or voluntary or conservation or infrastructure or care or streets or communal or Guerrilla)).ti,ab. | 402 |
| 19 | greenspace.ti,ab. | 25 |
| 20 | 18 or 19 | 425 |
| 21 | (urban adj3 (green$ or park$1 or parkland or garden$ or horticultur$ or wood$ or forest$ or botanical or arboretum or allotment$ or (open adj1 space))).ti,ab. | 579 |
| 22 | ((work$ or renewal or volunteer$ or voluntary or practical or regenerat$ or restor$ or maintain$ or care or enhance or preserve or creat$) and (urban or city or metropolis or town$) and (garden$ or park$1 or parkland or allotment$)).ti,ab. | 370 |
| 23 | *Cities/ and ((work$ or renewal or volunteer$ or voluntary or practical or regenerat$ or restor$ or maintain$ or care or enhance or preserve or creat$) and (garden$ or park$1 or parkland or allotment$)).ti,ab. | 5 |
| 24 | *Urban Health/ and (*Conservation of Natural Resources/ or *Voluntary Workers/) | 19 |
| 25 | 21 or 22 or 23 or 24 | 914 |
| 26 | ((garden$ or horticulture or allotment$ or botanical or arboretum) adj5 (kitchen or school$ or college$ or university or campus or hospital$ or prison$ or penitentiary or institution or urban or green$ or communit$ or communal or group$ or guerrilla or (bio adj1 diver$) or eco or ((grow or pick) adj3 your own))).ti,ab. | 595 |
| 27 | ((garden$ or horticulture or allotment$ or botanical or arboretum) adj5 (maintain$ or creat$ or culivat$ or enhance$ or preserve or voluntary or volunteer or conservation$ or participat$)).ti,ab. | 120 |
| 28 | Gardening/ and (*Conservation of Natural Resources/ or *Voluntary Workers/) | 13 |
| 29 | *Gardening/ and (kitchen or school$ or college$ or university or campus or hospital$ or prison$ or penitentiary or institution or urban or green$ or communit$ or communal or group$ or guerrilla or (bio adj1 diver$) or eco or maintain$ or creat$ or culivat$ or voluntary or volunteer or conservation$ or participat$).ti,ab. | 118 |
| 30 | 26 or 27 or 28 or 29 | 754 |
| 31 | ((communit$ adj5 (group$ or team$ or association$ or organisation or organization or participa$ or stakeholder$ or steward$ or trust$ or ranger$ or activit$)) and (garden$ or allotment$ or forest or (natural and environment) or conservation$)).ti,ab. | 363 |
| 32 | (communit$ and (work$ or renewal or volunteer$ or voluntary or practical or regenerat$ or restor$ or maintain$ or care or enhance$ or preserve or creat$ or activ$ or action$ or involve$) and ((natur$ adj3 environment$) or (environmental$ and conservation$))).ti,ab. | 479 |
| 33 | (((communit$ or local) adj5 (garden$ or park$ or green$ or greenspace or outdoor$ or outside$ or pavement$ or sidewalk$ or wood$ or allotment$ or lake$ or canal$ or river$)) and (work$ or renewal or volunteer$ or voluntary or practical or participat$ or regenerat$ or restor$ or maintain$ or enhance or preserve or creat$)).ti,ab. | 813 |
| 34 | 31 or 32 or 33 | 1556 |
| 35 | 11 or 17 or 20 or 25 or 30 or 34 | 8554 |
| 36 | exp animals/ not humans.sh. | 3785951 |
| 37 | 35 not 36 | 6941 |
| 38 | (clinical or surgery or surgical or cell or cells or laboratory or placebo or bladder or uterus or breast or gene or genes or genetic or bowel or liver or enzymes or viral or lymph or molecular).mp. | 9317419 |
| 39 | 37 not 38 | 4815 |
| 40 | limit 39 to english language | 4349 |
| 41 | limit 40 to yr="1990 -Current" | 3896 |

Hits: 3896

Notes: N/A

File Saved: Medline Endnote RIS 3896.txt

**Databases searched:**

**ASSIA(ProQuest); BIOSIS(ISI); British Education Index(ProQuest); British Nursing Index (ProQuest); CAB Abstracts(CAB Direct); Campbell Collaboration; Cochrane Public Health Specialized Register; DOPHER (EPPI); EMBASE(Ovid); ERIC(ProQuest); Global Health(Ovid); GreenFILE(EBSCO); HMIC(Ovid); IBSS (ProQuest); MEDLINE in Process(Ovid); MEDLINE (Ovid); OpenGrey; PsychINFO (Ovid); Social Policy and Practice(Ovid); SPORTDiscus; TRoPHI(EPPI); Social Services Abstracts (ProQuest); Sociological Abstracts(ProQuest); The Cochrane Library; TRIP database; and Web of Science (ISI).**

**S3. Data extraction forms**

**Table S3. Quantitative data**

| **Study details** | **Population** | **EECA description** | **Outcomes** | **Notes** |
| --- | --- | --- | --- | --- |
| **Authors:**  **Year published:**  **Publication type:**  **Year of research:**  **Title:**  **Aims:**  **Study design:**  **Analysis approach:**  **Funders:**  **Country:**  **Region:**  **Rural/urban:**  **Deprivation index:** | **Population included:**  **Participant characteristics:**   - **Sample size:** - **Age:** - **% Female:** - **Ethnicity:** - **Occupation:** - **Religion:** - **Education:** - **SEP:** - **Disability** - **Sexual orientation:** - **Vulnerable group:** - **Recruitment:**   **Control:**  **Other population notes:** | **Intervention description:**   - **Activity typology:** - **Engagement (voluntary/constrained/forced):** - **Training undertaken:**   **Time frame:**  **Frequency:**  **Provider:**  **Location in nature (Woodland/urban green space…):**  **Theoretical underpinning identified by the authors:**  **Formal:**  **Lay:**  **Theoretical underpinning identified by the review team:** | **Physical:**  **Mental:**  **Wellbeing:**  **Social:**  **Other:** | **Limitations of study as identified by authors:**  **Limitations of study as identified by review team:** |
| **Results** | | | | |
|  | | | | |

**Table S4. Qualitative data**

| **Study details** | **Population** | **EECA description** | **Method** | **Notes** |
| --- | --- | --- | --- | --- |
| **Authors:**  **Year published:**  **Year of research:**  **Title:**  **Aims:**  **Funders:**  **Country:**  **Region:**  **Rural/urban:**  **Deprivation index:** | **Population included:**   - **Sample size:** - **Age:** - **% Female:** - **Ethnicity:** - **Occupation:** - **Religion:** - **Education:** - **SEP:** - **Disability** - **Sexual orientation:** - **Vulnerable group:** - **Other SE variables:** - **Recruitment:**   **Other population notes:** | **Intervention description:**  **Time frame:**  **Frequency:**  **Provider:**  **Location in nature (Woodland/urban green space…):**  **Theoretical underpinning (of ACTIVITIES) identified by the authors:**  **Theoretical underpinning (of ACTIVITIES) identified by the review team:** | **Design details:**   - **Data collection method:** - **Sampling:** - **Analysis:**   **Researcher/reflexivity:**  **Description of analysis process:**  **Themes identified:** | **Limitations of study as identified by authors:**  **Limitations of study as identified by review team:** |
| **Results** | | | | |
| **Don’t forget:**   - **Key themes** - **Key concepts** - **Relevant quotes** | | | | |

**S4. Results flow diagram**

**Figure S1. Results flow diagram**


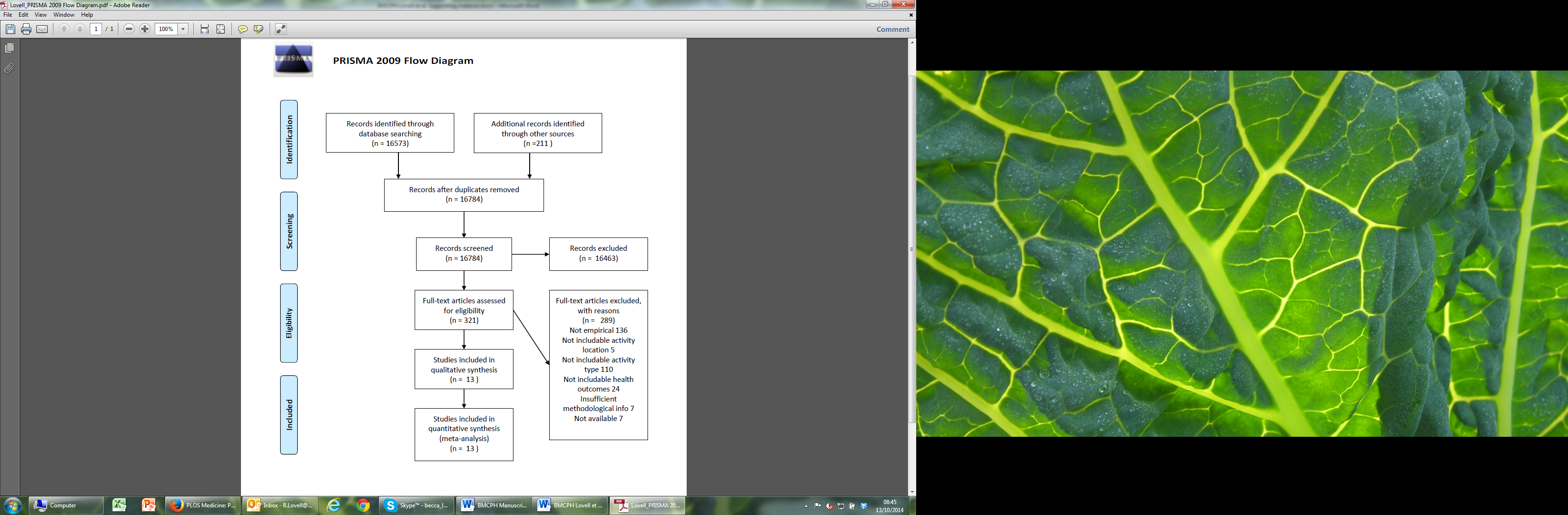


**S4. Quality assessments**

Table S5. Quantitative study quality

|  |  |  | Selection Bias | Study Design | Confounders | Blinding | Data Collection | Withdrawals and Dropouts |  |
| --- | --- | --- | --- | --- | --- | --- | --- | --- | --- |
|  |  |  |  |  |  |  |  |  |  |
|  |  |  |  |  |  |  |  |  |  |
|  |  |  |  |  |  |  |  |  |  |
|  |  |  |  |  |  |  |  |  |  |
|  |  |  |  |  |  |  |  |  |  |
|  |  |  |  |  |  |  |  |  |  |
|  |  |  |  |  |  |  |  |  |  |
|  | N | Design |  |  |  |  |  |  | Global rating** |
| Barton et al. 2009 | 19 | uBA | 2 | 3 | 2 | 3 | 1 | 3 | Weak |
| Brooker and Brooker 2008* | 1 | Nof1 | 3 | 3 | 3 | 3 | 3 | 3 | Weak |
| Brooker and Brooker 2008a* | 1 | Nof1 | 3 | 3 | 3 | 3 | 3 | 3 | Weak |
| BTCV 2009 | 122 | uBA | 3 | 3 | 3 | 3 | 3 | 1 | Weak |
| BTCV 2010b | 136 | uBA | 2 | 3 | 3 | 2 | 3 | 3 | Weak |
| Eastaugh et al. 2010 | 8 | uBA | 3 | 3 | 3 | 3 | 2 | 3 | Weak |
| O'Brien et al. 2010 | 88 | uBA | 3 | 3 | 2 | 3 | 3 | 1 | Weak |
| Pillemer et al. 2010 | 2630 | Cohort | 3 | 3 | 1 | 2 | 3 | 3 | Weak |
| Reynolds 1999a | 16 | uBA | 3 | 3 | 3 | 3 | 3 | 3 | Weak |
| Small Woods 2011 | 7 | uBA | 3 | 3 | 3 | 3 | 2 | 3 | Weak |
| Townsend et al. 2005 | 102 | Case-control | 3 | 2 | 1 | 2 | 3 | 1 | Weak |
| Wilson 2009 | 77 | uBA | 3 | 3 | 3 | 3 | 2 | 3 | Weak |
| 3: Weak; 2: Moderate; 1: Strong  * Not included in final synthesis | |  |  | | | |  |  |  |
| ** Global rating assigned according to EPHPP methodology | | | | | |  |  |  |  |


Table S6. Qualitative study quality.

|  |  |  | Research question clear? | Perspective of author clear? | Perpec. Influenced design? | Design appropriate? | Context described? | Sample adequate? | Sample drawn from app. pop.? | Data collection adeq. described? | Data coll. rigorously conducted? | Data analysis described? | Findings/limitations adequate? | Generalise from data? | Ethical issues addressed? |  |  |
| --- | --- | --- | --- | --- | --- | --- | --- | --- | --- | --- | --- | --- | --- | --- | --- | --- | --- |
|  |  |  |  |  |  |  |  |  |  |  |  |  |  |  |  |  |  |
|  |  |  |  |  |  |  |  |  |  |  |  |  |  |  |  |  |  |
|  |  |  |  |  |  |  |  |  |  |  |  |  |  |  |  |  |  |
|  |  |  |  |  |  |  |  |  |  |  |  |  |  |  |  |  |  |
|  |  |  |  |  |  |  |  |  |  |  |  |  |  |  |  |  |  |
|  |  |  |  |  |  |  |  |  |  |  |  |  |  |  |  |  |  |
|  |  |  |  |  |  |  |  |  |  |  |  |  |  |  |  |  |  |
|  | N | Design |  |  |  |  |  |  |  |  |  |  |  |  |  | Overall rating | Pass E_D? |
| Birch 2005 | 3 | Interviews | Y | Y | Y | Y | Y | N | Y | Y | Y | Y | Y | Y | Y | Poor | N |
| BTCV 2010 | 19 | Interviews | N | N | Y | Y | Y | Y | Y | Y | CT | Y | N | Y | N | Poor | N |
| Burls 2007 | 11 | Focus Groups | N | Y | Y | Y | Y | CT | CT | N | CT | CT | Y | Y | CT | Poor | N |
| Carter and O'Brien 2008 | NR | "Qual evaluation" | N | Y | CT | CT | Y | CT | CT | N | CT | CT | N | Y | N | Poor | N |
| Christie 2004 | 18 | Interviews | Y | Y | Y | Y | Y | Y | Y | Y | CT | CT | N | Y | N | Poor | N |
| Gooch 2005 | 85 | Interviews | Y | N | CT | Y | N | CT | CT | Y | CT | Y | Y | CT | N | Poor | N |
| Halpenny and Caissie 2003 | 10 | Interviews | Y | Y | Y | Y | Y | Y | Y | Y | Y | Y | Y | Y | Y | Good | Y |
| Miller et al. 2002 | 30 | Focus Groups, ethnographic | CT | N | CT | Y | Y | CT | Y | Y | CT | Y | Y | CT | Y | Poor | N |
| O'Brien et al. 2010 | 88 | Interviews | Y | Y | Y | Y | Y | Y | Y | Y | Y | Y | Y | Y | N | Good | Y |
| O'Brien et al. 2011 | 10 | Ethnographic | Y | Y | CT | Y | Y | Y | CT | Y | Y | Y | Y | CT | Y | Good | Y |
| Townsend and Marsh 2004 | 18 | Interviews | Y | Y | Y | Y | Y | CT | CT | N | CT | CT | N | CT | N | Poor | N |
| Townsend 2006 | 35 | Interviews | N | Y | Y | Y | Y | CT | CT | N | CT | CT | Y | N | N | Poor | N |
| Wilson 2009 | 29 | Interviews | Y | CT | CT | Y | Y | Y | Y | N | CT | Y | Y | CT | Y | Poor | N |

Y- Yes, N- No, CT – can’t tell.

Studies were rated ‘Poor’ if they did not achieve a yes in each of the 6 essential criteria (highlighted in red).


**S5. Supplementary evidence details**

**Table S7. Supplementary evidence details**

| Theme | Reference | Reference title | Type of research | Size | Date | Setting | Population | Results (quoted from abstract) |
| --- | --- | --- | --- | --- | --- | --- | --- | --- |
| Physical Activity | Arsland et al. 2010 [67] | Is physical activity a potential preventive factor for vascular dementia? A systematic review | Systematic review | 24 longitudinal studies | post 1990 | Not given | Adults (18+yrs) | The majority of individual studies did not report significant associations. Five studies fulfilled criteria for meta-analysis, including 10,108 non-demented control subjects and 374 individuals with VaD. The meta-analysis demonstrated a significant association between physical exercise and a reduced risk of developing VaD: OR 0.62 (95% CI 0.42–0.92). |
| Physical Activity | Ballard-Barbash et al. 2012 [62] | Physical Activity, Biomarkers, and Disease Outcomes in Cancer Survivors: A Systematic Review | Systematic review | 45 studies: 27 observational studies, 13 RCTs | 1950-2011 | Not given | Not given | Physical activity is associated with reduced all-cause, breast cancer–specific, and colon cancer–specific mortality. There is currently insufficient evidence regarding the association between physical activity and mortality for survivors of other cancers. Randomized controlled trials of exercise that included biomarker endpoints suggest that exercise may result in beneficial changes in the circulating level of insulin, insulin-related pathways, inflammation, and, possibly, immunity; however, the evidence is still preliminary |
| Physical Activity | Barnett et al. 2011 [55] | Physical activity and the transition to retirement: A mixed-method systematic review. Barnet | Systematic review | 24 studies: 19 quantitative and 5 qualitative | majority post 2000 | majority USA and industrialised countries | Older adults | Recreational physical activity increased with retirement, but overall physical activity (including occupational, transport, home and recreational) decreased. In retirees from lower socioeconomic groups, physical activity declined, whereas it increased in people from higher socioeconomic groups. In the qualitative studies, participants emphasised the importance of physical activity after retirement. Health benefits and opportunities to build new social support networks were common reasons to be physically active. Regular physical activity also helped to establish new daily structures and provided ‘a new purpose in life’ for many after leaving the labour force. Barriers to physical activity were lack of motivation, health problems, costs, caregiving responsibilities and perceived inappropriateness of physical activity in older age. Retirees from manual occupations were concerned about the unproductiveness of recreational physical activities. |
| Physical Activity | Bize et al, 2007 [56] | Physical activity level and health-related quality of life in the general adult population: a systematic review | Systematic review | 14 studies: seven were cross-sectional studies, two were cohort studies, four were randomized controlled trials and one used a combined cross sectional and longitudinal design | majority post 2002 | Not given | Adults | Cross-sectional studies showed a consistently positive association between self-reported physical activity and health-related quality of life. The largest cross-sectional study reported an adjusted odds ratio of "having 14 or more unhealthy days" during the previous month to be 0.40 (95% Confidence Interval 0.36-0.45) for those meeting recommended levels of physical activity compared to inactive subjects. Cohort studies and randomized controlled trials tended to show a positive effect of physical activity on health-related quality of life, but similar to the cross-sectional studies, had methodological limitations |
| Physical Activity | Blake et al. 2009 [68] | How effective are physical activity interventions for alleviating depressive symptoms in older people? A systematic review | Systematic review | 11 RCTs | 1966-2008 (specific dates dependant on database) | Not given | >60yrs | Short-term positive outcome for depression or depressive symptoms was found in nine studies, although the mode, intensity and duration of intervention varied across studies. Medium- to long-term effects of intervention were less clear. |
| Physical Activity | Eijkemans et al. 2012 [66] | Physical Activity and Asthma: A Systematic Review and Meta-Analysis | Systematic review | 39 studies: 5 longitudinal studies and 34 cross-sectional studies | to 2011 | majority western countries | All | Pooling of the longitudinal studies showed that subjects with higher physical activity levels had lower incidence of asthma (odds ratio 0.88 (95% CI: 0.77–1.01)). When restricting pooling to the 4 prospective studies with moderate to good study quality (defined as NOS≥5) the pooled odds ratio only changed slightly (0.87 (95% CI: 0.77–0.99)). |
| Physical Activity | Ferreira et al. 2012 [65] | Physical activity improves strength, balance and endurance in adults aged 40-65 years: a systematic review | Systematic review | 23 RCTs (17 in pooled meta-analysis) | to 2010 | Not given | 40-65 yrs ('healthy') | The meta-analysis of strength outcomes found a moderate effect of physical activity on strength (SMD=0.54, 95% CI 0.38 to 0.70). Larger effects were observed from programs that specifically targeted strength (SMD=0.68, 95% CI 0.49 to 0.87), when compared to those that did not (SMD=0.32, 95% CI 0.09 to 0.55). This difference was statistically significant (effect of strength in meta-regression p=0.045). Physical activity also had a moderate effect on both balance (SMD=0.52, 95% CI 0.24 to 0.79) and endurance (SMD=0.73, 95% CI 0.50 to 0.96). No trials reported effects of physical activity on falls soon after receiving the intervention. A statistically non-significant effect on falls 15 years after receiving a physical activity intervention was found in one trial (RR=0.82, 95% CI 0.53 to 1.26) |
| Physical Activity | Hamilton et al. 2010 [64] | The effects of exercise and physical activity participation on bone mass and geometry in postmenopausal women: a systematic review of pQCT studies | Systematic review | 12 studies: 4 RCTs, 1 nonRCT, 3 cross-sectional and 4 prospective cohort studies | to 2008 | Not given | postmenopausal women | Exercise effects appear to be modest, site-specific, and preferentially influence cortical rather than trabecular components of bone. Exercise type also plays a role, with the most prominent mass and geometric changes being observed in response to high-impact loading exercise. Exercise appears to positively influence bone mass and geometry in postmenopausal women |
| Physical Activity | Janssen. 2010 [59] | Systematic review of the health benefits of physical activity and fitness in school-aged children and youth | Systematic review | 86 cross-sectional studies, case-control studies, cohort studies (prospective and retrospective) and intervention studies (including randomized and quasi experimental designs) - specific breakdown not given | post 1950 (specific date dependant on database) | Not given | School aged children and youth (5-17 yrs) | Physical activity was associated with numerous health benefits. The dose-response relations observed in observational studies indicate that the more physical activity, the greater the health benefit. Results from experimental studies indicate that even modest amounts of physical activity can have health benefits in high-risk youngsters (e.g., obese). To achieve substantive health benefits, the physical activity should be of at least a moderate intensity. Vigorous intensity activities may provide even greater benefit. Aerobic-based activities had the greatest health benefit, other than for bone health, in which case high-impact weight bearing activities were required. |
| Physical Activity | Jimenez-Pavon et al. 2010 [57] | Associations between objectively measured habitual physical activity and adiposity in children and adolescents: Systematic review | Systematic review | 48 studies: majority cross-sectional | 2004-2008 | Not given | Children and adolescents | There was consistent evidence of negative associations between objectively measured physical activity and adiposity: significant negative associations were observed in 38/48 (79%) of studies overall. |
| Physical Activity | Kay et al. 2006 [58] | The influence of physical activity on abdominal fat: a systematic review of the literature | Systematic review | 27 studies: Nineteen randomized controlled trials (RCTs) and eight non-randomized controlled trials were selected. | 1985-2005 | Not given | 18+ (excluded HIV positive populations, included Diabetes Type 2) | In RCTs using imaging techniques to measure change in abdominal fat in overweight or obese subjects, seven out of 10 studies (including three trials with type 2 diabetics) reported significant reductions compared with controls. Reductions in visceral and total abdominal fat may occur in the absence of changes in body mass and waist circumference. Waist-to-hip ratio is not a sensitive measure of change in regional adiposity in exercise studies |
| Physical Activity | Penedo et al. 2005 [61] | Exercise and well-being: a review of mental and physical health benefits associated with physical activity |  |  |  |  |  |  |
| Physical Activity | Spence et al. 2010 [69] | Exercise and cancer rehabilitation: a systematic review | Systematic review | 10 studies (including RCT, controlled clinical trial (non-randomised) and randomised trials | to 2009 | Not given | Adult (16+) | Improvements in physical functioning, strength, physical activity levels, quality of life, fatigue, immune function, haemoglobin concentrations, potential markers of recurrence, and body composition were reported |
| Physical Activity | Windle et al. 2010 [60] | Is exercise effective in promoting mental well-being in older age? A systematic review | Systematic review | 13 studies | 1993-2007 | Not given | Living at home, in the community, in supported housing or in residential care homes | An overall effect of exercise on mental well-being was found (standardised effect size = 0.27; CI = 0.14-0.40). The included interventions were designed for older people, targeted those who are sedentary and delivered in a community setting, primarily through a group-based approach led by trained leaders. |
| Physical Activity | Woodcock et al. 2011 [63] | Non-vigorous physical activity and all-cause mortality: systematic review and meta-analysis of cohort studies | Systematic review | 22 Cohort studies | no time restrictions | Not given | Adult (studies with n>10,000) | A 2.5 h/week (equivalent to 30 min daily of moderate intensity activity on 5 days a week) compared with no activity was associated with a reduction in mortality risk of 19% [95% confidence interval (CI) 15–24], while 7 h/week of moderate activity compared with no activity reduced the mortality risk by 24% (95% CI 19–29). We found a smaller effect in studies that looked at walking alone |
| Achievement | Choi et al. 2013 [72] | Participation in productive activities and depression among older Europeans: Survey of Health, Ageing and Retirement in Europe (SHARE) | Cross-sectional study | 7238 participants | 2004/5 and 2006/7 | 14 European countries | relatively healthy community residents aged 60 years and older' | Depression was less prevalent among those individuals who were employed or self-employed and those who participated in formal volunteering or informal helping, whereas caregiving was associated with a higher risk of depression. Caring for grandchildren was not associated positively or negatively with depression. Formal volunteering and caregiving remained associated with depression after adjustment for age, sex, marital status, education, economic status, country and presence of long-term illness |
| Achievement | Feinstein and Hammond . 2004 [77] | The contribution of adult learning to health and social capital | Longitudinal study | 11500 participants | 1991 and 2000 | UK | age 33 and 42 | Adult learning plays an important role in contributing to the small shifts in attitudes and behaviours that take place during mid‐adulthood. The results hold as controls are added for demographic, educational and other background factors, as well as for changes in life circumstances during mid‐adulthood. It is therefore very likely that there are substantive and genuine effects of adult learning. However, we do not suggest a purely one‐way causal relationship. Evidence from additional analyses suggests rather that participation in adult learning is a very important element in positive cycles of development and progression. |
| Achievement | Hammond. 2004 [76] | Impacts of lifelong learning upon emotional resilience, psychological and mental health: fieldwork evidence | Qualitative - biographical interviews | 145 participants | Not given | UK | Adults | Participation in lifelong learning had effects upon a range of health outcomes; wellbeing, protection and recovery from mental health difficulties, and the capacity to cope with potentially stress-inducing circumstances including the onset and progression of chronic illness and disability. These effects were mediated by relatively immediate impacts of learning upon psychosocial qualities; self-esteem, self-efficacy, a sense of purpose and hope, competences, and social integration. Learning developed these psychosocial qualities through extending boundaries, a process which is quintessential to learning. However, not all educational experiences had positive effects upon health outcomes. Provision that generated positive health outcomes matched the interests, strengths and needs of the learner |
| Achievement | Hatch et al. 2007 [78] | The Continuing Benefits of Education: Adult Education and Midlife Cognitive Ability in the British 1946 Birth Cohort | Longitudinal study | 1934 participants | 1999 | UK | 8, 26, 36, 43, and 53 yrs | Educational attainment completed by early adulthood was associated with all measures of cognitive ability in late midlife. The continued effect of education was apparent in the associations between adult education and higher verbal ability, verbal memory, and verbal fluency in late midlife. We found no association between adult education and mental speed and concentration. Associations between adult education and midlife cognitive ability indicate wider benefits of education to health that may be important for social integration, well-being, and the delay of cognitive decline in later life |
| Achievement | Headety. 2008 [70] | Life Goals Matter to Happiness: A Revision of Set-Point Theory | Longitudinal and cross-sectional panel survey | 3553 longitudinal, 20,000 cross-sectional | 1990,1995,2004,2005 longitudinal. 2004 Cross-sectional | Germany | 16+ | A high level of life satisfaction is quite strongly associated with giving high priority to family goals, and significantly associated with high priority for altruistic goals. Goals relate in theoretically interesting and plausible ways to life satisfaction. Life goals matter substantially to subjective well-being (SWB). Non-zero sum goals, which include commitment to family, friends and social and political involvement, promote life satisfaction. Zero sum goals, including commitment to career success and material gains, appear detrimental to life satisfaction. Finding that conscious life goals matter can potentially make an important contribution to SWB theory. The most widely accepted theory – set-point or dynamic equilibrium theory – essentially claims that set-points are near-automatic consequences of hereditary characteristics, including personality traits. Life goals play no role in these theories and major life events are viewed as having only a transitory effect. |
| Achievement | Huppert. 2008 [71] | Psychological Wellbeing: Evidence regarding its causes and consequences–State-of-Science Review: SR-X2 | Strategic review | N/A | Not given | Global | All | Extensive research, both experimental and observational, on goal pursuit shows that enhanced subjective wellbeing is associated with: goals being intrinsic i.e. self-generated; progress towards a valued goal; the pursuit of approach goals rather than avoidance goals; and the pursuit of goals congruent with personal values. In addition, a large body of work shows that active participation in social activities and involvement in one’s community is associated with high levels of happiness and life satisfaction. An intentional activity, that is activities over which we have control, are also very important drivers of psychological wellbeing. These researchers divide intentional activities into three broad groups: (a) behaviours – such as taking regular exercise or being kind to others; (b) cognitions – such as interpreting events in a positive light or feeling gratitude; and (c) motivations – such as striving towards goals that reflect deeply- held values rather than being driven by external rewards. |
| Achievement | Onyx and Warburton. 2003 [74] | Volunteering and health among older people: a review | Review (semi-systematic) | Not given | 1997-2007 | Focus on Australia and western developed world | All | There was consistent evidence that morbidity rates, functional health indices, self-reported health and life satisfaction are affected by formal and informal volunteering. Some studies suggest that the benefits of volunteering are reciprocal, in that both those who give and those who receive assistance benefit. The evidence is consistent with the proposal that social capital is generated through volunteering. It is likely that the presence of high levels of social capital supports and maintains the health of older persons, provides informal support in times of sickness and stress and thus enhances quality of life as well as reducing or delaying the onset of illness and death. |
| Achievement | Von Bonsdoff and Rantanen. 2011 [73] | Benefits of formal voluntary work among older people. A review | Narrative review | 16 studies | Not given | USA | >60 years | Volunteering in old age predicted better self-rated health, functioning, physical activity and life satisfaction as well as decreased depression and mortality. However, it did not decrease the risk of chronic diseases or nursing home admission in old age. |
| Social Contact | Egan et al. 2008 [82] | Psychosocial risk factors in home and community settings and their associations with population health and health inequalities: A systematic meta-review | Systematic meta-review | 31 reviews | Not given | Not given | Not given | Some evidence was found to show favourable psychosocial environments associated with better health. Reviews also found evidence of unfavourable psychosocial risk factors linked to poorer health, particularly among socially disadvantaged groups. However, the more robust reviews each identified studies with inconclusive findings, as well as studies finding evidence of associations. We also identified some evidence of apparently favourable psychosocial risk factors associated with poorer health. |
| Social Contact | Holt-Lundstadt. 2010 [79] | Social Relationships and Mortality Risk: A Meta-analytic Review | Systematic review | 143 studies | 1900-2007 | (participants) 51% from North America, 37% from Europe, 11% from Asia, and 1% from Australia | Not given | A 50% increased likelihood of survival for participants with stronger social relationships. This finding remained consistent across age, sex, initial health status, cause of death, and follow-up period. Significant differences were found across the type of social measurement evaluated (*p*<0.001); the association was strongest for complex measures of social integration (OR = 1.91; 95% CI 1.63 to 2.23) and lowest for binary indicators of residential status (living alone versus with others) (OR = 1.19; 95% CI 0.99 to 1.44). |
| Social Contact | Nyqvist et al. 2012 [80] | Social capital as a resource for mental well-being in older people: A systematic review | Systematic review | 11 studies | 1990-2011 | not given | >50 yrs | All included studies found positive associations between parts of social capital and aspects of mental well-being. Typically, the relationship between social capital and mental well-being differed within as well as between studies. Social capital is generated in the interaction between individual and collective life. |
| Social Contact | Steptoe et al. 2013 [81] | Social isolation, loneliness, and all-cause mortality in older men and women | longitudinal study | 6500 participants | 2004-5 and 2012 | England | >52 years | Mortality was higher among more socially isolated and more lonely participants. However, after adjusting statistically for demographic factors and baseline health, social isolation remained significantly associated with mortality (hazard ratio 1.26, 95% confidence interval, 1.08–1.48 for the top quintile of isolation), but loneliness did not (hazard ratio 0.92, 95% confidence interval, 0.78–1.09). The association of social isolation with mortality was unchanged when loneliness was included in the model. Both social isolation and loneliness were associated with increased mortality. However, the effect of loneliness was not independent of demographic characteristics or health problems and did not contribute to the risk associated with social isolation. |
| Natural environment | Annerstedt and Wahrborg. 2011 [89] | Nature-assisted therapy: Systematic review of controlled and observational studies | Systematic review | 38 studies (3 systematic reviews and meta-analyses, 6 randomised controlled trials, 12 non-randomised intervention trials, 14 observational studies, and 4 qualitative studies) | 1980-2009 | Not given | patients with a disease as defined by the International Classification of Diseases (ICD 10, International Classification of Diseases, version 2007, WHO), or otherwise a well-defined state of ill health | For the studies with high evidence grade, the results were generally positive, though somewhat ambiguous. Among the studies of moderate to low evidence grade, health improvements were reported in 26 cases out of 29. This review gives at hand that a rather small but reliable evidence base supports the effectiveness and appropriateness of NAT as a relevant resource for public health. Significant improvements were found for varied outcomes in diverse diagnoses, spanning from obesity to schizophrenia |
| Natural environment | Bowler et al. 2010 [2] | A systematic review of evidence for the added benefits to health of exposure to natural environments | Systematic review | 28 studies | Not given | Not given | All | Some evidence was identified to show favourable psychosocial environments associated with better health. Reviews also found evidence of unfavourable psychosocial risk factors linked to poorer health, particularly among socially disadvantaged groups. However, the more robust reviews each identified studies with inconclusive findings, as well as studies finding evidence of associations. We also identified some evidence of apparently favourable psychosocial risk factors associated with poorer health. |
| Natural environment | Di Nardo et al. 2012 [88] | Green areas and health outcomes: a systematic review of the scientific literature | Systematic review | 15 studies (one review, one case-control study, 3 cohort studies and 8 cross-sectional studies) | to 2006 | Not given | All | The reported findings were generally consistent and supported the current view that urban design and the availability of urban green spaces are key elements of prosperity and individual/collective comfort, so as to influence both the perceived health and the objective physical conditions in a measurable way. A weak relationship between physical activity levels and green space availability is observed. |
| Natural environment | Lachywycx and Jones. 2011 [87] | Greenspace and obesity: a systematic review of the evidence | Systematic review | 60 studies | 2000-2010 | Not given | Not given | The majority (68%) of papers found a positive or weak association between greenspace and obesity-related health indicators, but findings were inconsistent and mixed across studies. Several studies found the relationship varied by factors such as age, socioeconomic status and greenspace measure. |
| Natural environment | Lee and Maheswaran. 2011 [86] | The health benefits of urban green spaces: a review of the evidence | Systematic review | 35 studies | 1990-2010 | high-income countries | Not given | There is weak evidence for the links between physical, mental health and well-being, and urban green space. Environmental factors such as the quality and accessibility of green space affects its use for physical activity. User determinants, such as age, gender, ethnicity and the perception of safety, are also important. |
| Natural environment | Thompson-Coon et al. 2011 [1] | Does participating in physical activity in outdoor natural environments have a greater effect on physical and mental wellbeing than physical activity indoors? A systematic review | Systematic review | 11 controlled trials | to 2010 | Not given | All | Most trials (n = 9) showed some improvement in mental wellbeing on one or other of the outcome measures. Compared with exercising indoors, exercising in natural environments was associated with greater feelings of revitalization and positive engagement, decreases in tension, confusion, anger, and depression, and increased energy. However, the results suggested that feelings of calmness may be decreased following outdoor exercise. Participants reported greater enjoyment and satisfaction with outdoor activity and declared a greater intent to repeat the activity at a later date. None of the identified studies measured the effects of physical activity on physical wellbeing or the effect of natural environments on exercise adherence. |
